# Multiancestry brain pQTL fine-mapping and integration with genome-wide association studies of 21 neurologic and psychiatric conditions

**DOI:** 10.1038/s41588-025-02291-2

**Published:** 2025-09-08

**Authors:** Aliza P. Wingo, Yue Liu, Selina M. Vattathil, Ekaterina S. Gerasimov, Zhen Mei, Suda Parimala Ravindran, Jiaqi Liu, Ananth Shantaraman, Fatemeh Seifar, Erming Wang, Bin Zhang, Joseph Reddy, Mariet Allen, Nilüfer Ertekin-Taner, Philip L. De Jager, Edward J. Fox, Duc M. Duong, Michael P. Epstein, David J. Cutler, Allan I. Levey, David A. Bennett, Nicholas T. Seyfried, Thomas S. Wingo

**Affiliations:** 1 VA Northern California Healthcare System, Mather, CA USA; 2 Department of Psychiatry, School of Medicine, University of California, Davis, Sacramento, CA USA; 3 Department of Neurology, School of Medicine, University of California, Davis, Sacramento, CA USA; 4 Department of Psychiatry, Emory University, Atlanta, GA USA; 5 Department of Biochemistry, Emory University School of Medicine, Atlanta, GA USA; 6 Center for Neurodegenerative Disease, Emory University School of Medicine, Atlanta, GA USA; 7 Department of Genetics and Genomic Sciences, Icahn School of Medicine at Mount Sinai, New York, NY USA; 8 Mount Sinai Center for Transformative Disease Modeling, Icahn School of Medicine at Mount Sinai, New York, NY USA; 9 Department of Quantitative Health Sciences, Mayo Clinic Florida, Jacksonville, FL USA; 10 Department of Neuroscience, Mayo Clinic Florida, Jacksonville, FL USA; 11 Department of Neurology, Mayo Clinic Florida, Jacksonville, FL USA; 12 Center for Translational and Computational Neuroimmunology, Department of Neurology, Columbia University Irving Medical Center, New York, NY USA; 13 Department of Human Genetics, Emory University School of Medicine, Atlanta, GA USA; 14 Department of Neurology, Emory University School of Medicine, Atlanta, GA USA; 15 Goizueta Alzheimer’s Disease Research Center, Emory University School of Medicine, Atlanta, GA USA; 16 Rush Alzheimer’s Disease Center, Rush University Medical Center, Chicago, IL USA; 17 Alzheimer’s Disease Research Center, School of Medicine, University of California, Davis, Sacramento, CA USA

**Keywords:** Alzheimer's disease, Depression, Gene expression profiling, Proteome informatics

## Abstract

To understand shared and ancestry-specific genetic control of brain protein expression and its ramifications for disease, we mapped protein quantitative trait loci (pQTLs) in 1,362 brain proteomes from African American, Hispanic/Latin American and non-Hispanic white donors. Among the pQTLs that multiancestry fine-mapping MESuSiE confidently assigned as putative causal pQTLs in a specific population, most were shared across the three studied populations and are referred to as multiancestry causal pQTLs. These multiancestry causal pQTLs were enriched for exonic and promoter regions. To investigate their effects on disease, we modeled the 858 multiancestry causal pQTLs as instrumental variables using Mendelian randomization and genome-wide association study results for neurologic and psychiatric conditions (21 traits in participants with European ancestry, 10 in those with African ancestry and 4 in Hispanic participants). We identified 119 multiancestry pQTL–protein pairs consistent with a causal role in these conditions. Remarkably, 29% of the multiancestry pQTLs in these pairs were coding variants. These results lay an important foundation for the creation of new molecular models of neurologic and psychiatric conditions that are likely to be relevant to individuals across different genetic ancestries.

## Abstract (web summary)

Multiancestry fine-mapping of brain protein quantitative trait loci coupled with Mendelian randomization analyses identifies protein–trait pairs consistent with causal effects across neurological and psychiatric conditions.

## Main

A major goal of human genetics is to understand how genetic variation influences human traits and diseases. The most straightforward path involves genetic variation affecting RNA and protein expression, which in turn influences traits. To date, most genetic studies of both RNA and protein expression have used samples from participants of European ancestry. However, populations of different ancestries can have differences in linkage disequilibrium (LD), allele frequency or genetic effects^1^, which can influence the accuracy of cross-population estimates of genetic risk such as polygenic risk scores (PRS)^1^ or identification of risk genes^2^. For instance, when a reference genome-wide association study (GWAS) was conducted in participants of European ancestry and the test population was not of European ancestry, the prediction of disease using the estimated PRS was only 20–40% as accurate as that for a European ancestry test population^1^. As another example, risk genes for a disorder can be identified by integrating GWAS results with reference gene expression data in a transcriptome-wide association study (TWAS)^3^. A TWAS using a GWAS of African American (AA) participants and transcriptomes from European Americans showed a notable reduction in prediction accuracy compared to a TWAS using both a GWAS and transcriptomes from European Americans^2^.

Findings of substantial differences in PRS and TWAS predictions across ancestries suggest the possibility of important differences in genetic contributions to disease between these ancestries. This could have profound effects on the potential to develop therapeutic agents that can be broadly applied across individuals of different ancestries. On the other hand, these differences might be primarily driven by mundane factors such as allele frequency and LD differences. Distinguishing the profoundly important from the mundane is only possible if high-confidence ‘causal’ variants can be found. Here, ‘causal’ variants refer to genetic variants that drive the variation in RNA or protein expression among expression quantitative trait loci (eQTLs) or protein quantitative trait loci (pQTLs). If such variants are known, we can test whether they act similarly in multiple ancestry groups independently of LD and allele frequency to address whether there are substantial differences in genetic effects between different ancestries. In the absence of their identification, it remains an open question whether apparent differences in eQTLs or pQTLs are due to differences in genetic effects versus allele frequency or LD.

**Fig. 1:**
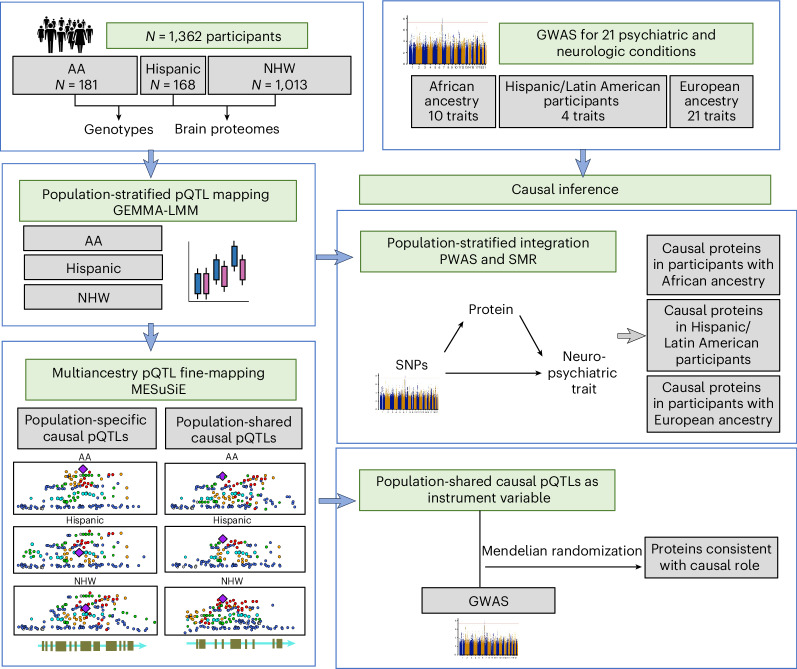
Study design. We performed population-stratified pQTL mapping, multiancestry pQTL fine-mapping and causal inference.

In light of that, we here examined the genetic control of brain protein expression in 1,362 diverse donors comprising AA, Hispanic/Latin American and non-Hispanic white (NHW) individuals. We mapped pQTLs in each population separately using proteomic profiles from the dorsolateral prefrontal cortex (Fig. 1). Then we applied multiancestry pQTL fine-mapping to identify putative causal pQTLs that were shared or specific to a population. Next, we examined the role of the population-stratified pQTLs and multiancestry causal pQTLs in the pathogenesis of 21 neurologic and psychiatric disorders. Collectively, these results shed light on whether causal pQTLs are shared across ancestries and greatly expand the number of proteins consistent with a causal role in 21 brain conditions. Of the multiancestry causal pQTLs we found to be involved in the pathogenesis of these conditions, 29% were in coding variants. These findings lay an important foundation for the creation of new molecular models for these polygenic neurologic and psychiatric disorders that are likely to be relevant to individuals across different ancestries.

## Results

### Population-stratified brain pQTL mapping

Using mass spectrometry, we profiled brain proteomes from the dorsolateral prefrontal cortex of 1,362 multiancestry donors that included AA, Hispanic and NHW (Supplementary Table 1). Groups were initially identified using self-assigned labels and compared to genetically defined clustering to reduce genetic heterogeneity within each group. A total of 11,748 proteins were detected, and proteomic profiles underwent rigorous quality control and normalization to remove effects of batch, postmortem interval (PMI), age, sex, diagnosis and surrogate variables before pQTL mapping. After quality control of genetic and proteomic data, 8,884 proteins remained for AA ancestry, 8,648 for Hispanic ancestry and 9,254 for NHW ancestry for pQTL mapping. We performed *cis*-pQTL mapping in each population separately, adjusting for sex using a linear mixed model (LMM)^4^, which inherently accounted for sample relatedness and population substructure. After clumping (at LD *r*^2^ < 0.1), we identified 7,852 independent candidate pQTLs in the AA population, 7,499 in the Hispanic population and 40,087 in the NHW population (Supplementary Table 2a), consistent with the corresponding sample size and statistical power. The genomic inflation factors for these pQTL mappings were similar to those of published plasma pQTL mapping studies^5,6^ (Supplementary Table 2b). Compared with the largest published brain pQTL study in individuals of European ancestry (*n* = 716)^7^, the current study found 49% more brain pQTLs and 28% new genes with a pQTL (pGenes) with its larger sample size (Supplementary Note A). We further investigated whether some pQTLs were present in the AA population but absent from the NHW population and vice versa (Methods) and found that 12.8% of the AA candidate pQTLs were not NHW candidate pQTLs, and 1.1% of the NHW candidate pQTLs were not AA candidate pQTLs owing to allele frequency differences.

### Multiancestry pQTL fine-mapping

To identify pQTLs that were likely to drive variation in protein expression and determine whether they were shared or specific to populations, we performed multiancestry pQTL fine-mapping with MESuSiE^8^. The MESuSiE framework accounts for variation in LD in different ancestries and leverages association information across ancestries by using summary statistics from the population-stratified pQTL mapping as input^8^. MESuSiE explicitly models both shared and population-specific single-nucleotide polymorphisms (SNPs) and considers only SNPs that are present in all the examined populations at minor allele frequency (MAF) > 0.05, as well as allowing for multiple causal variants per locus^8^. MESuSiE provides two probabilities related to the confidence of a SNP being a causal pQTL: the posterior inclusion probability (PIP), which represents the confidence that a SNP is a causal pQTL in any of the studied populations; and the categorical PIP (category.PIP), which represents the probability that a pQTL is a causal pQTL in a specific set of populations.

A total of 8,498 proteins were present in all three populations for multiancestry pQTL fine-mapping. MESuSiE identified 5,133 95% credible sets that likely contained causal pQTLs in at least one population for 3,336 unique genes (Supplementary Table 3). Notably, there were fewer SNPs in these credible sets than in those from single-ancestry fine-mapping with NHW data using SuSiE^9^ (median 6 (interquartile range (IQR) 2–27) versus 9 (IQR 3–24); Welch’s *t*-test *P* = 9.2 × 10^−15^), suggesting an improvement in fine-mapping resolution with multiancestry data.

**Fig. 2:**
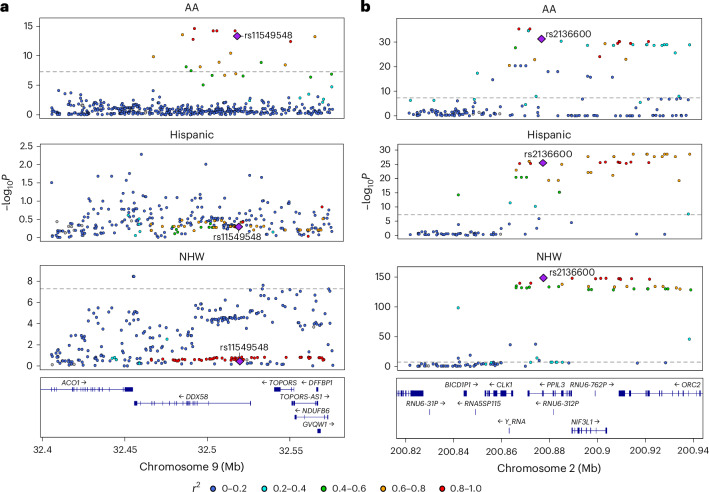
Causal pQTLs specific to or shared among populations. **a**,**b**, LocusZoom plots of two loci identified as causal pQTLs specific to a population (**a**) and shared among three populations (**b**) based on MESuSiE pQTL fine-mapping results: the *DDX58* locus on chromosome 9 for each population (**a**) and the *PPIL3* locus on chromosome 2 for each population (**b**). Tested SNPs within each locus are plotted by position on the *x* axis and −log *P* value on the *y* axis for the association with the abundance of the respective protein. The putative causal site is labeled and denoted by a purple diamond, and each tested SNP is colored by its ${r}^{2}$ with the causal site. The statistical test used was the restricted likelihood ratio test with multiple testing correction, followed by the Bayesian framework. Full lists of these causal pQTLs are provided in Supplementary Tables 3 and 4.

Multiancestry fine-mapping in the three studied populations with MESuSiE yielded seven possible categories: (1) shared across AA, Hispanic and NHW; (2) shared between AA and NHW; (3) shared between AA and Hispanic; (4) shared between Hispanic and NHW; (5) specific to AA; (6) specific to Hispanic; and (7) specific to NHW. To examine putative causal pQTLs that were shared or specific to a population, we used a threshold of category.PIP > 50% and found 1,367 putative causal pQTLs assigned to one of these categories (Supplementary Table 4). From these, we further filtered out those that had discordant direction of association or were not nominally significant in the population-stratified pQTL mapping and obtained 1,131 putative causal pQTLs at category.PIP > 0.5 (Table 1). A large majority of the causal pQTLs were shared across all three populations. Specifically, at category.PIP > 50%, 75.9% of the causal pQTLs were shared across all three populations (Table 1 and Supplementary Table 4). At category.PIP > 90%, 99.5% of the causal pQTLs were shared across all three populations (Table 1). We refer to the causal pQTLs shared across all three populations at category.PIP > 0.5 as putative multiancestry causal pQTLs. The 858 putative multiancestry causal pQTLs corresponded to 797 unique genes (pGenes; Supplementary Table 5). Figure 2a shows the LocusZoom plot of an example causal pQTL that was specific to AA (rs11549548 in *DDX58*) and Fig. 2b depicts a multiancestry causal pQTL (rs2136600 in *PPIL3*).

**Table 1:** Shared and population-specific putative causal pQTLs from multiancestry pQTL fine-mapping with MESuSiE

			Causal pQTLs per category						
			Shared across populations				Population-specific		
		AA	●	●	●		●		
		NHW	●	●		●		●	
Category.PIP threshold	Total causal pQTLs	Hisp	●		●	●			●
>50%	1,131		858 (75.9%)	70 (6.2%)	0	180 (15.9%)	3 (0.3%)	20 (1.8%)	0
>75%	521		510 (97.9%)	4 (0.8%)	0	7 (1.3%)	0	0	0
>90%	373		371 (99.5%)	1 (0.3%)	0	1 (0.3%)	0	0	0

Category.PIP in MESuSiE represents the confidence that a causal pQTL is assigned to one of the seven potential configurations of population groupings. For three populations, the seven possible configurations were: (1) shared across AA, Hispanic and NHW; (2) shared between AA and NHW; (3) shared between AA and Hispanic; (4) shared between NHW and Hispanic; (5) specific to AA; (6) specific to NHW; and (7) specific to Hispanic. The total causal pQTLs column indicates the total number of causal pQTLs at that category.PIP threshold, and the remaining columns give the numbers of causal pQTLs assigned to each group configuration. Full results are provided in Supplementary Table 4.

To determine whether sample size differences among the studied populations influenced the number of pQTLs shared among populations or specific to a population, we downsampled the NHW proteomic dataset from 1,013 to 191 to make its size comparable to the sample sizes of the AA (*n* = 181) and Hispanic (*n* = 168) populations (Methods). Consistently, we found that most of the causal pQTLs were shared across all three populations with the smaller NHW proteomic dataset (82.8% and 98.9% were multiancestry causal pQTLs at category.PIP > 0.5 and category.PIP > 0.9, respectively; Supplementary Table 6). Together, these findings suggest that the great majority of the putative causal pQTLs detectable at sample sizes in the low hundreds are shared across the populations.

We estimated the ${\pi }_{1}$ replication rate for these 858 multiancestry causal pQTLs in each of the three underlying populations. The ${\pi }_{1}$ statistic estimates the proportion of the multiancestry causal pQTLs that are also pQTLs in each population. We found that ${\pi }_{1}$ rates exceeded 99% in each of the three populations. Next, we examined the consistency of the pQTL effect by calculating pairwise correlations of the beta values from ancestry-stratified pQTL mapping for the 858 multiancestry causal pQTLs. We found all pairwise correlations (AA versus NHW, AA versus Hispanic and NHW versus Hispanic) to be ≥0.95 (all *P* < 2.2 × 10^−16^), suggesting a high level of consistency in effect sizes. In addition, we compared our brain pQTLs with three published plasma pQTLs using the Olink and SomaScan platforms. Replication was generally high, considering differences in tissues, proteomic technology and sample sizes, lending confidence to our findings of putatively shared causal pQTLs (Supplementary Note A).

To understand the biological relevance of the 858 putative multiancestry causal pQTLs, we examined their genomic-site-type annotations and compared them with those among the candidate pQTLs identified in the population-stratified pQTL mapping. We found that these multiancestry causal pQTLs were significantly more enriched than the candidate pQTLs for promoter regions (8% versus 5%), untranslated regions (UTRs; 13% versus 8%), synonymous sites (2% versus 1%) and nonsynonymous sites (13% versus 5%) (Supplementary Table 7).

### Cross-ancestry protein prediction

**Extended Data Fig. 1:**
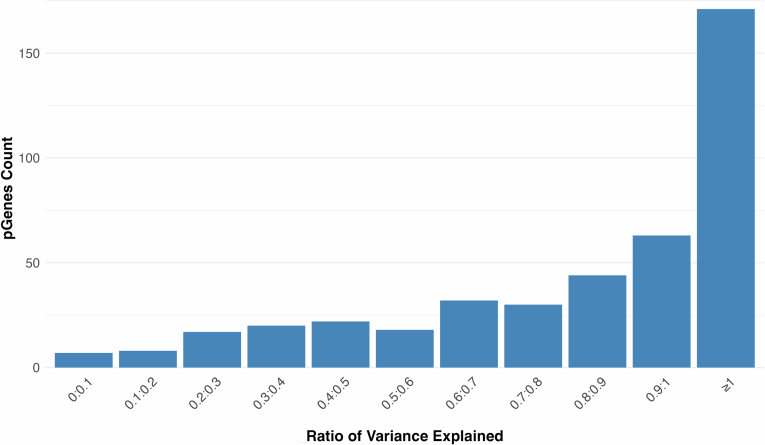
Cross-ancestry prediction of genetically regulated protein expression for the pGenes corresponding to the 858 multi-ancestry causal pQTLs. The plot depicts accuracy of cross-ancestry prediction of genetically regulated protein abundance for the pGenes corresponding to the 858 multi-ancestry causal pQTLs. The *x*-axis shows the ratio of the variance explained (R^2^) of cross-ancestry to same-ancestry prediction. Higher ratios indicate more accuracy. The *y*-axis reflects the number of pGenes for a particular ratio bin. The data underlying this plot are provided as Source Data. Source data

To evaluate the accuracy of cross-ancestry prediction of genetically regulated protein abundance for the pGenes corresponding to the 858 multiancestry causal pQTLs, we performed cross-ancestry prediction for these pGenes in the AA population using NHW data (Methods and Supplementary Table 8). The cross-ancestry prediction *R*^2^ had a median of 0.15 (s.d.: 0.12; maximum: 0.61), where a higher *R*^2^ value indicates more variance of protein abundance explained by genetically regulated protein expression. The single-ancestry prediction (using AA proteomic and genetic data to predict genetically regulated protein levels in the AA population) had a median cross-validation *R*^2^ of 0.19 (s.d.: 0.14; maximum: 0.69) for these pGenes (Supplementary Table 8). To compare the cross-ancestry with single-ancestry prediction, we took the ratio of the cross-ancestry *R*^2^ and the same-ancestry *R*^2^ for 432 heritable pGenes present in both models. We found that approximately 69% of these pGenes had a ratio >0.75, suggesting reasonable cross-ancestry prediction for most pGenes with multiancestry causal pQTLs (Extended Data Fig. 1).

### Integration of brain proteomes with GWASs

To understand how genetic variation contributes to neurologic and psychiatric disorders through brain protein expression, we integrated population-matched pQTLs with GWAS results for 21 neurologic and psychiatric disorders (21 GWASs in participants of European ancestry, 10 in those of African ancestry and 4 in Hispanic participants; Supplementary Table 9). For each trait and in each population separately, we first performed a brain proteome-wide association study (PWAS) to identify brain proteins whose genetically regulated expression levels were associated with the trait of interest using proteomes from the same ancestry as the GWAS using the FUSION pipeline^3^. We performed Mendelian randomization for the genes identified by the PWAS using SMR^10^ to pinpoint brain proteins that were causal mediators of the GWAS signals for each trait in each population separately using population-matched pQTLs and GWAS results. The HEIDI test^10^ was applied to filter out results that were likely due to LD (that is, HEIDI *P* < 0.05). Taking into consideration findings from the PWAS, SMR and HEIDI, we defined proteins contributing to the pathogenesis of a neurologic or psychiatric condition as those meeting all of the following criteria: (1) being identified as a significant protein in the PWAS (PWAS false discovery rate (FDR) < 0.05); (2) being a significant protein according to the Mendelian randomization test (SMR *P* < 0.05); (3) the significant association in SMR not being due to LD (HEIDI *P* > 0.05); and (4) directions of association between protein and trait being in a consistent direction between the PWAS and SMR. We refer to these as candidate causal proteins.

We identified one candidate causal protein for Parkinson’s disease (GBA1) and two for alcohol use disorder (METAP1 and ADH5) in participants of African ancestry (Table 2 and Supplementary Table 10). Notably, the statistical power in our integration approach depends largely on the statistical power of the GWAS and moderately on the sample size of the proteomes; therefore, the smaller sample sizes and consequent lower power of the GWAS in African and Hispanic populations yielded a limited number of causal proteins in these populations. No causal proteins were identified in the Hispanic population. In the NHW population, for psychiatric conditions, we identified 216 candidate causal proteins for major depression, 170 for bipolar disorder, 257 for schizophrenia, 31 for post-traumatic stress disorder (PTSD), 10 for anxiety, 44 for attention-deficit hyperactivity disorder (ADHD), 2 for opioid addiction, 30 for insomnia, 2 for cannabis use disorder, 125 for neuroticism, 76 for tobacco use, 10 for anorexia and 55 for alcohol use disorder (Table 2 and Supplementary Table 11). For neurologic conditions, among NHW participants, we found 64 candidate causal proteins for Alzheimer’s disease, 1 for Lewy body dementia, 10 for stroke, 18 for Parkinson’s disease and 16 for amyotrophic lateral sclerosis (Table 2 and Supplementary Table 11). Among the three candidate causal proteins identified in participants of African ancestry, one was also a causal protein in the NHW population for the corresponding trait (GBA1 for Parkinson’s disease), but the other two were causal in the AA population only (METAP1 and ADH5 in alcohol use disorder), indicating that these are potential population-specific causal proteins.

**Table 2:** Candidate causal proteins in neurologic and psychiatric conditions from integration of population-matched pQTLs and GWAS

NHW				
			Number of significant proteins	
	GWAS *n*	Percentage of cases in GWAS	PWAS	PWAS and SMR/HEIDI
Alcohol use disorder	753,000	15%	90	55
Alzheimer’s disease	789,000	14%	91	64
Amyotrophic lateral sclerosis	138,000	20%	20	16
Anorexia	73,000	23%	20	10
Anxiety	175,000	–	13	10
ADHD	226,000	17%	56	44
Bipolar disorder	413,000	10%	252	170
Cannabis use disorder	358,000	4%	2	2
Insomnia	387,000	–	42	30
Lewy body dementia	7,000	40%	1	1
Major depressive disorder	1,350,000	27%	334	216
Neuroticism	390,000	–	180	125
Opioid addiction	488,000	7%	2	2
Parkinson’s disease	1,474,000	4%	25	18
PTSD	214,000	17%	37	31
Schizophrenia	131,000	41%	392	257
Stroke	1,308,000	6%	17	10
Tobacco use	326,000	–	118	76

African ancestry				
			Number of significant proteins	
	GWAS cases *n*	Percentage of cases in GWAS	PWAS	PWAS and SMR/HEIDI
Alcohol use disorder	123,000	33%	2	2
Parkinson’s disease	4,000	33%	2	1
Stroke	24,000	17%	2	0

Results of integration of population-matched brain pQTLs and GWASs using PWASs and SMR/HEIDI for the NHW and AA populations for a range of traits. The sample sizes and proportions of cases in each GWAS are given for each trait except for anxiety, insomnia, neuroticism and tobacco use, which were treated as quantitative variables. Proteins consistent with a causal role in the trait were identified based on a significant PWAS (FDR < 0.05), a significant SMR/HEIDI result (SMR *P* < 0.05 and HEIDI *P* > 0.05) and a consistent direction of effect between PWASs and SMR. The numbers of causal proteins are listed in the last column. No proteins were significant in the analyses in the Hispanic population. Full results are provided in Supplementary Tables 10 and 11.

As a secondary analysis, we integrated population-matched brain proteomic results with GWAS results in each ancestry and/or ethnicity separately for these 21 neurologic and psychiatric conditions using PMR-Egger^11^, a probabilistic Mendelian randomization framework that tests and controls for horizontal pleiotropy^11^. By design, PMR-Egger uses correlated SNPs as instrumental variables, similar to the PWAS approach, and thus is analogous to the PWAS/SMR approach described above. We found that although the PWAS/SMR approach was more conservative, it was less prone to false positive findings (Supplementary Note B and Supplementary Tables 12–16).

### Multiancestry causal pQTLs underlying brain conditions

**Fig. 3:**
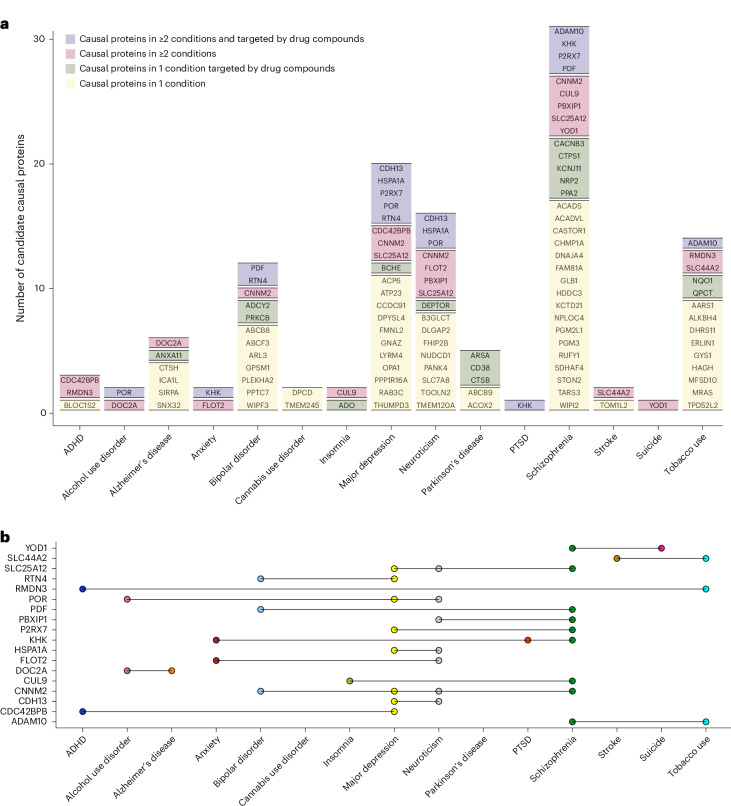
Candidate causal proteins in neurologic and psychiatric conditions derived from multiancestry causal pQTLs using Mendelian randomization. Using the 858 multiancestry causal pQTLs as the instrument variable, we performed Mendelian randomization using population-matched GWASs. Proteins with SMR FDR < 0.05 and HEIDI *P* > 0.05 were considered to be candidate causal proteins. In total, 119 multiancestry causal pQTL–protein–trait triads were found in the NHW population. None was found in the AA or Hispanic population, likely owing to smaller GWASs with limited power. Fifteen of the 21 tested brain conditions had multiancestry causal pQTL–protein–trait triads. **a**, Bar plot listing the causal proteins for each trait on the horizontal axis. Colors denote whether the protein was identified as causal for two or more conditions or targeted by an existing drug compound. **b**, Upset plot illustrating traits for the causal proteins identified as causal for ≥2 traits. For example, POR is a causal protein in alcohol use disorder targeted by existing drug compounds; it is also causal in major depression, and neuroticism. Detailed results are provided in Supplementary Table 17.

Guided by the finding that most of the putative causal pQTLs were shared across the studied populations, making them broadly applicable to all studied populations, we used the 858 multiancestry causal pQTLs as instrumental variables in Mendelian randomization to identify pQTL–protein pairs contributing to the pathogenesis of these 21 brain conditions using results from each GWAS. Through SMR^10^, we identified 119 multiancestry causal pQTL–protein–trait triads in the NHW population (Fig. 3 and Supplementary Table 17) but none in either African or Hispanic participants, likely owing to the lower GWAS sample sizes for these ancestries. For neurologic disorders, we found eight multiancestry causal pQTL–protein pairs for Alzheimer’s disease, five for Parkinson’s disease and two for stroke (Fig. 3 and Supplementary Table 17). For psychiatric disorders, we found 2 multiancestry causal pQTL–protein pairs for alcohol use disorder, 2 for anxiety, 4 for ADHD, 14 for bipolar disorder, 3 for cannabis use disorder, 2 for insomnia, 21 for major depressive disorder, 16 for neuroticism, 1 for PTSD, 32 for schizophrenia and 14 for tobacco use (Fig. 3 and Supplementary Table 17). These 119 multiancestry causal pQTL–protein pairs are likely to be causal in all three populations as they were derived with multiancestry causal pQTLs as the genetic instrumental variable.

Notably, approximately one-third (40 of 119) of the multiancestry causal pQTL in these triads were in coding regions (24% nonsynonymous and 7.6% in the 3′ UTR or 5′ UTR; Table 3 and Supplementary Table 17). Furthermore, 20% (18 of 96) of the multiancestry causal pQTL–protein pairs were implicated in one or more traits, consistent with pleiotropy among the traits. For example, the rs1140239–DOC2A pair was causal in both alcohol use disorder and Alzheimer’s disease, consistent with epidemiologic observations that problematic alcohol use is a risk factor for dementia^12^. Likewise, rs1057868–POR was implicated in neuroticism, major depression and alcohol use disorder, which frequently co-occur. Together, these findings suggest promising therapeutic targets and potential entry points for new model systems for mechanistic study of these complex neuropsychiatric disorders.

**Table 3:** Multiancestry causal pQTLs contributing to neurologic and psychiatric conditions and located in exons

Gene symbol	rsID	Freq.	Exonic variant location or coding change	Trait	pQTL		MESuSiE		SMR		HEIDI
					*β* (s.e.)	*P*	PIP	Category.PIP	*β* (s.e.)	*P*	*P*
*ADO*	rs2236295	0.4	NM_032804:c.73G>T (p.Gly25Trp)	Insomnia	−1.00 (0.038)	6.26 × 10^−150^	0.96	0.96	−0.02 (0.005)	8.03 × 10^−06^	0.53
*ALKBH4*	rs41275227	0.17	NM_017621:c.740C>T (p.Ala247Val)	Tobacco use	−0.88 (0.054)	6.33 × 10^−60^	1.00	1.00	0.01 (0.003)	6.21 × 10^−04^	0.34
*ARSA*	rs2071421	0.13	NM_001085427:c.1055A>G (p.Asn352Ser)	Parkinson’s disease	−1.20 (0.060)	3.28 × 10^−90^	1.00	1.00	0.08 (0.021)	3.56 × 10^−04^	0.90
*CUL9*	rs2273709	0.20	NM_015089:c.6173A>C (p.His2058Pro)	Insomnia	−0.35 (0.058)	1.40 × 10^−09^	NA	0.84	−0.10 (0.025)	2.62 × 10^−05^	0.09
				Schizophrenia	−0.35 (0.058)	1.40 × 10^−09^			0.16 (0.041)	7.07 × 10^−05^	0.18
*DEPTOR*	rs4871827	0.33	NM_022783:c.1166G>A (p.Ser389Asn)	Neuroticism	0.63 (0.046)	3.16 × 10^−43^	1.00	0.60	−0.02 (0.004)	8.02 × 10^−05^	0.63
*DLGAP2*	rs2301963	0.45	NM_001346810:c.1391C>A (p.Pro464Gln)	Neuroticism	−0.48 (0.044)	4.21 × 10^−28^	0.67	0.67	−0.02 (0.005)	1.61 × 10−04	0.14
*DOC2A*	rs1140239	0.38	NM_003586:c.142G>A (p.Gly48Ser)	Alcohol use disorder	0.65 (0.044)	9.77 × 10^−51^	0.61	0.58	−0.02 (0.004)	7.67 × 10^−07^	0.13
				Alzheimer’s disease	0.65 (0.044)	9.77 × 10^−51^			−0.09 (0.014)	3.98 × 10^−10^	0.07
*ERLIN1*	rs2862954	0.48	NM_006459:c.871A>G (p.Ile291Val)	Tobacco use	−0.79 (0.040)	4.15 × 10^−87^	0.52	0.52	0.01 (0.003)	3.78 × 10^−06^	0.31
*FMNL2*	rs4664114	0.34	synonymous	Major depressive disorder	−0.38 (0.046)	1.01 × 10^−16^	0.97	0.53	0.03 (0.009)	1.26 × 10^−03^	0.17
*KCNJ11*	rs5219	0.38	NM_000525:c.67A>G (p.Lys23Glu)	Schizophrenia	−0.38 (0.046)	1.44 × 10^−16^	0.72	0.64	0.08 (0.026)	1.25 × 10^−03^	0.19
*KCTD21*	rs613686	0.16	3′ UTR	Schizophrenia	−1.18 (0.055)	1.59 × 10^−102^	1.00	1.00	0.04 (0.010)	1.57 × 10^−04^	0.78
*KHK*	rs2304681	0.37	NM_006488:c.145G>A (p.Val4Ile)	Anxiety	−0.96 (0.042)	6.34 × 10^−116^	1.00	1.00	0.03 (0.006)	2.01 × 10^−05^	0.59
				PTSD	−0.96 (0.042)	6.34 × 10−116			0.25 (0.051)	1.38 × 10^−06^	0.34
				Schizophrenia	−0.96 (0.042)	6.34 × 10^−116^			0.04 (0.009)	1.85 × 10^−05^	0.19
*LYRM4*	rs2224391	0.24	NM_020408:c.31T>G (p.Ser11Ala)	Major depressive disorder	−1.03 (0.046)	2.69 × 10^−113^	1.00	1.00	0.01 (0.003)	1.17 × 10^−03^	0.54
*NPLOC4*	rs3208787	0.15	3′ UTR	Schizophrenia	−0.33 (0.062)	1.19 × 10^−07^	1.00	0.75	0.15 (0.047)	2.07 × 10^−03^	0.90
*OPA1*	rs1056366	0.39	3′ UTR	Major depressive disorder	0.49 (0.044)	3.47 × 10^−28^	0.82	0.60	−0.03 (0.006)	5.31 × 10^−05^	0.08
*P2RX7*	rs3751143	0.19	NM_002562:c.1445A>C (p.Glu496Ala)	Major depressive disorder	−1.12 (0.050)	7.66 × 10^−112^	1.00	1.00	−0.01 (0.003)	4.77 × 10^−05^	0.49
				Schizophrenia	−1.12 (0.050)	7.66 × 10^−112^			−0.04 (0.010)	1.43 × 10^−04^	0.14
*PANK4*	rs7535528	0.39	NM_018216:c.1640C>T (p.Ala547Val)	Neuroticism	−0.93 (0.040)	1.28 × 10^−116^	1.00	1.00	−0.01 (0.003)	4.95 × 10^−05^	0.36
*PBXIP1*	rs2061690	0.43	NM_020524:c.1070G>A (p.Gly357Asp)	Neuroticism	−0.55 (0.042)	5.11 × 10^−38^	0.68	0.67	−0.02 (0.004)	2.13 × 10^−04^	0.47
				Schizophrenia	−0.55 (0.042)	5.11 × 10^−38^			−0.06 (0.017)	4.20 × 10^−04^	0.07
*PDF*	rs8057004	0.31	NM_022341:c.31T>C (p.Trp11Arg)	Bipolar disorder	0.64 (0.045)	1.12 × 10^−44^	0.97	0.71	0.07 (0.016)	5.12 × 10^−05^	0.39
				Schizophrenia	0.64 (0.045)	1.12 × 10^−44^			0.06 (0.015)	1.90 × 10^−04^	0.20
*PGM2L1*	rs12049823	0.16	NM_173582:c.41T>C (p.Leu14Pro)	Schizophrenia	0.89 (0.058)	9.64 × 10^−54^	0.57	0.56	−0.05 (0.014)	1.67 × 10^−04^	0.52
*POR*	rs1057868	0.28	NM_001395413:c.1499C>T (p.Ala500Val)	Alcohol use disorder	0.55 (0.047)	1.06 × 10^−31^	0.68	0.65	−0.02 (0.005)	2.15 × 10^−06^	0.25
				Major depressive disorder	0.55 (0.047)	1.06 × 10^−31^			−0.02 (0.006)	9.85 × 10^−05^	0.12
				Neuroticism	0.55 (0.047)	1.06 × 10^−31^			−0.02 (0.005)	1.23 × 10^−04^	0.05
*PPA2*	rs4699179	0.21	Synonymous	Schizophrenia	−0.68 (0.053)	6.68 × 10^−37^	0.65	0.65	0.06 (0.016)	5.04 × 10^−04^	0.06
*STON2*	rs2241621	0.42	NM_001394390:c.2722T>G (p.Ser908Ala)	Schizophrenia	0.52 (0.043)	2.07 × 10^−33^	0.93	0.93	0.05 (0.017)	2.94 × 10^−03^	0.22
*TGOLN2*	rs4247303	0.50	NM_006464:c.775C>T (p.Arg259Trp)	Neuroticism	−0.53 (0.043)	5.13 × 10^−34^	1.00	1.00	0.02 (0.004)	5.48 × 10^−04^	0.62
*THUMPD3*	rs11547584	0.19	5′ UTR	Major depressive disorder	−0.64 (0.055)	4.42 × 10^−32^	0.97	0.81	0.02 (0.006)	6.07 × 10^−04^	0.13
*TOM1L2*	rs7501812	0.38	3′ UTR	Stroke	−0.85 (0.042)	2.57 × 10^−91^	1.00	1.00	−0.04 (0.008)	2.92 × 10^−05^	0.76
*TPD52L2*	rs8567	0.47	3′ UTR	Tobacco use	0.54 (0.042)	1.98 × 10^−38^	1.00	0.99	0.01 (0.004)	1.37 × 10^−04^	0.07
*WIPI2*	rs13244634	0.24	3′ UTR	Schizophrenia	−0.55 (0.050)	3.49 × 10^−28^	1.00	0.99	0.06 (0.019)	2.63 × 10^−03^	0.40
*YOD1*	rs1044145	0.44	3′ UTR	Schizophrenia	−0.75 (0.041)	2.05 × 10^−74^	1.00	1.00	0.05 (0.012)	3.81 × 10^−05^	0.80
				Suicide	−0.75 (0.041)	2.05 × 10^−74^			0.06 (0.014)	4.12 × 10^−06^	0.24

These are the multiancestry causal pQTL–trait pairs identified by Mendelian randomization using the 858 multiancestry shared causal pQTLs as the instrumental variables. We list here the multiancestry causal pQTLs located in exons, including nonsynonymous, synonymous pQTLs and those in 3′ or 5′ UTRs. For nonsynonymous pQTLs, the coding change with respect to the canonical transcript is provided. Eight genes (*CUL9*, *DOC2A*, *KHK*, *P2RX7*, *PBXIP1*, *PDF*, *POR* and *YOD1*) are causal for multiple traits. Full results are provided in Supplementary Table 17. Wald test was used. Multiple testing was adjusted for with the FDR. Freq., minor allele frequency.

### Molecular processes, drug targets and drug repurposing

**Fig. 4:**
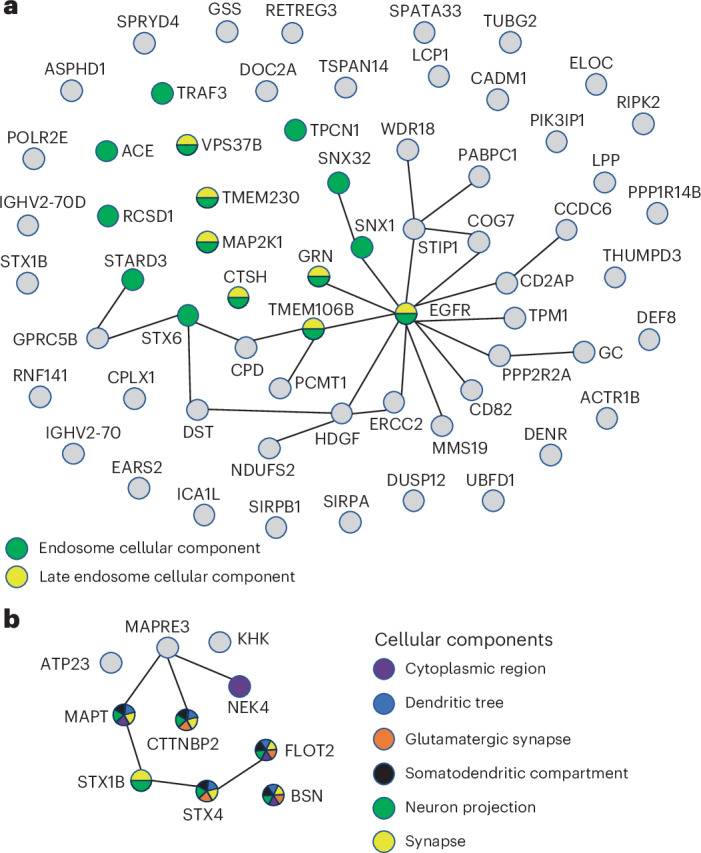
Causal proteins and their physical PPIs and gene set enrichment results for Alzheimer’s disease and anxiety symptoms. Candidate causal proteins were identified through population-matched integration of brain proteomes and GWASs using PWASs and SMR/HEIDI. Each gene is plotted as a circle, with the color denoting an enriched process (gray indicates genes not in an enriched gene set). Solid lines connecting the genes denote evidence of PPI. **a**, For Alzheimer’s disease, 64 causal proteins were identified. Of these, 29 had evidence of PPIs among themselves, and there was gene set enrichment for endosomal proteins (FDR < 0.05). **b**, For anxiety symptoms, ten causal proteins were identified, and seven had PPIs with each other. Some of these anxiety causal proteins were enriched in cellular components of the cytoplasmic region, dendritic tree, somatodendritic neuron projection, synapse and glutamatergic synapse (FDR < 0.05). Full results are provided in Supplementary Tables 18 and 19.

The 1,140 candidate causal proteins identified through integration of human brain proteogenomic data with GWAS results for 21 neurologic and psychiatric conditions provide insight into mechanisms underlying the pathogenesis of these brain conditions and represent promising targets for therapeutic development. We examined physical protein–protein interactions (PPIs) among the candidate causal proteins for each condition and found that many interacted with each other (Supplementary Table 18). For instance, in Alzheimer’s disease, 29 of 64 candidate causal proteins had physical PPIs between or among each other, with the number of PPIs per protein ranging from 1 to 12 (Fig. 4a). As another example, 7 of 10 anxiety candidate causal proteins interacted with each other, with a range of 1–3 PPIs per protein (Fig. 4b and Supplementary Table 18). Through gene set enrichment analysis, we found that candidate causal proteins in Alzheimer’s disease were enriched for genes involved in endosomal processes (Fig. 4a and Supplementary Table 19), those in schizophrenia for cellular components of the glutamatergic synapse, and those in anxiety for cellular components of the dendritic tree, somatodendritic compartment and the synapse, and for neuron projection and glutamatergic neurotransmission (Fig. 4b and Supplementary Table 19).

When possible, drug repurposing can offer a faster and often more cost-effective alternative to de novo drug development. We used the Drug Gene Interaction Database^13^ to identify available drug compounds that target these candidate causal proteins. Of the 1,140 protein–trait pairs identified in the NHW population (Table 2), approximately 25% were targeted by drug compounds that had been tested in clinical trials, and many of these drug compounds had been approved for treatment of a medical condition (Supplementary Tables 20 and 21). In addition, approximately one-third of the 119 multiancestry causal pQTL–protein–trait triads were targeted by at least a drug compound that had been tested in clinical trials (Fig. 3 and Supplementary Tables 20 and 22). Together, these candidate causal proteins lay an important foundation for therapeutic development and drug repurposing efforts.

## Discussion

This study examined shared and ancestry-specific genetic control of brain protein expression using genetic and brain proteomic profiles from AA, Hispanic and NHW donors. Through multiancestry pQTL fine-mapping that accounted for differences in allele frequency and LD between populations, we identified putative causal pQTLs and determined whether they were shared or specific to a population. Notably, we found that at category.PIP > 0.75, the vast majority of the putative causal pQTLs (97.9%) were shared across the three studied populations. At category.PIP > 0.5, 76% of the causal pQTLs were shared across all three populations and are referred to as multiancestry causal pQTLs. These multiancestry causal pQTLs showed striking enrichment for functional genomic regions beyond that observed for population-stratified candidate pQTLs, especially for UTRs and nonsynonymous coding sites. This is noteworthy because no functional information was used for fine-mapping in the MESuSiE framework. Thus, although there remains the potential for ancestry-specific differences in genetic effects on protein abundance, nearly all high-confidence causal pQTLs we identified showed little to no evidence of ancestry differences. Together, these findings suggest that a great majority of the putative causal pQTLs detectable at sample sizes in the low hundreds are shared across the populations.

We observed at least three advantages to incorporating other ancestries in pQTL fine-mapping. First, multiancestry pQTL fine-mapping yielded credible sets with higher resolution than NHW-only fine-mapping. Second, multiancestry fine-mapping identified more causal pQTLs and had a higher confidence level for the identified causal pQTLs. This can be illustrated by examining the PIP for the 858 multiancestry causal pQTLs in MESuSiE and SuSiE; the median and mean PIP for these in MESuSiE were 0.97 and 0.87, respectively, compared to 0.49 and 0.53, respectively, for SuSiE. Moreover, only 471 of the 858 pQTLs were identified as causal pQTLs in SuSiE (Supplementary Table 23). Thus, multiancestry fine-mapping affords more confidence and precision in identification of causal pQTLs. Third, we identified two candidate causal proteins specific to individuals of African ancestry in alcohol use disorder (ADH5 and METAP1) despite lower statistical power from the corresponding GWAS and reference proteogenomic data. Larger GWASs and proteogenomic data from participants of African ancestry might yield more ancestry-specific causal proteins in neuropsychiatric conditions.

We used two approaches to investigate how genetic control of brain protein expression contributes to the pathogenesis of 21 neurologic and psychiatric conditions. In the first approach, we integrated population-matched GWASs with brain proteomes using PWASs and Mendelian randomization SMR. We identified 1,140 proteins consistent with a causal role in the 21 psychiatric and neurologic conditions; most of these were from the NHW populations, because GWASs in African ancestry and Hispanic populations tend to have lower sample sizes and consequently lower statistical power, highlighting the need for larger GWASs in these populations. Recognizing the limitation of statistical power in identifying causal proteins that could be generalizable to multiple genetic ancestries, we used a new tactic based on the finding that most causal pQTLs were shared across populations. Thus, in the second approach, we used the 858 multiancestry causal pQTLs as instrumental variables to perform Mendelian randomization using population-matched GWASs in each population separately to identify causal proteins. We identified 119 multiancestry causal pQTL–protein–trait triads for the neurologic and psychiatric disorders, corresponding to 96 unique causal proteins. Notably, 23.5% of the multiancestry causal pQTLs in these triads were nonsynonymous variants, and 7.6% were in the 3′ UTR or 5′ UTR. These 119 multiancestry causal pQTL–protein–trait triads are likely to be applicable to individuals of diverse ancestries for two main reasons. First, the multiancestry causal pQTLs were used as the instrumental variable in the Mendelian randomization performed to find these triads. Second, emerging evidence suggests that genetic causal effects on complex traits are similar across segments of different continental ancestries in admixed individuals^14^.

Historically, exonic coding variants have illuminated our understanding of the pathophysiology of many rare monogenic human illnesses (including cystic fibrosis and sickle cell anemia)^15^ or rare forms of common human disorders (including early-onset Alzheimer’s disease^16^ and early-onset Parkinson’s disease^17^). In light of this, we posit that studying multiancestry causal pQTL–protein–trait triads with functional pQTL variants is likely to help provide insight into the pathogenesis of the corresponding neurologic or psychiatric conditions. Beyond the opportunity to adapt the causal pQTL variants to disease-relevant models, we found that there were already drug compounds targeting about one-third of all of the multiancestry causal pQTL–protein–trait triads, suggesting drug repurposing opportunities.

The 1,140 candidate causal proteins identified through integration of human brain proteogenomic data with GWAS results of 21 neurologic and psychiatric conditions highlight and expand on constituents of disease-relevant pathways and could provide new insights into the molecular underpinnings of these neurologic and psychiatric conditions. We describe those in Alzheimer’s disease as an example in the Supplementary Note C. More studies are needed to elucidate how these risk proteins contribute to the pathogenesis of the corresponding psychiatric and neurologic conditions.

Interpretation of our findings should take into consideration the limitations of the study. Although we leveraged the largest human brain proteomes from diverse donors available at the time, we were best powered to detect signals that were shared among different populations. Post hoc power analysis showed that for the three causal pQTLs categorized as AA-specific at category.PIP > 0.5, there was at least 60% power to detect them in NHW and Hispanic groups at FDR < 0.05. For the 20 putative causal pQTLs categorized as NHW-specific at category.PIP > 0.5, several of them had low power in the AA and Hispanic groups. However, when we increased the confidence level to category.PIP > 75%, MESuSiE found no population-specific brain pQTLs. It is possible that shared causal pQTLs have, on average, larger effect sizes than population-specific ones, meaning that we were only well powered to find those that were shared at current sample sizes. Future work with larger sample sizes for AA and Hispanic brain proteomes may identify more population-specific pQTLs. Likewise, the numbers of causal proteins for neurologic and psychiatric conditions identified among Hispanic participants and those of African ancestry were limited largely by the smaller sample size in their GWASs and moderately by the number of brain proteomes; future studies with larger GWASs are likely to identify more causal proteins. In addition, the brain proteomes were from postmortem tissues, which represent the proteins at older age and may not be ideal for the study of psychiatric conditions that manifest in earlier life stages.

Our study also had several strengths. First, we performed multiancestry brain pQTL mapping and fine-mapping. Second, we followed a multiancestry fine-mapping approach that explicitly modeled both shared and population-specific causal pQTLs and accounted for different LD and allele frequencies in different populations. Third, we used proteomes profiled by tandem mass tag mass spectrometry, which is a highly reliable and reproducible untargeted proteomic platform^18^; moreover, we detected approximately 11,750 proteins, more than has been possible with other proteomic technologies^18^. Fourth, this is the largest integration to date of GWASs and brain proteomes in diverse populations using population-matched data to provide mechanistic insights into 21 neurologic and psychiatric conditions. Future work ought to focus on expanding the diversity and number of brain proteomes.

In summary, our findings suggest that the great majority of causal pQTLs in human brain are shared across ancestries and ethnicities and are highly enriched for functional variants. Furthermore, in addition to identifying hundreds of candidate causal proteins for the 21 neurologic and psychiatric conditions, we identified 119 multiancestry causal pQTL–protein–trait triads that are likely to be causal across all three populations and represent promising targets for the development of therapeutics for these neurologic or psychiatric disorders. Importantly, 29% of the multiancestry causal pQTLs were coding variants; thus, they represent an essential foundation for the creation of new molecular models for polygenic neurologic and psychiatric disorders that are likely to be relevant to individuals across ancestral backgrounds.

## Methods

### Ethics

Our study complies with all relevant ethical regulations and was approved by the institutional review boards at University of California, Davis; Rush University; the Mayo Clinic; Mount Sinai University; Emory University and Banner Sun Health Research Institute.

### Genetic data

Genetic data were generated from blood or brain tissue using either array-based genotyping or whole-genome sequencing (WGS) as described previously^19–22^. New to this study was WGS from 181 AA, 168 Hispanic and 292 NHW individuals whose raw Illumina 150-bp reads were mapped to GRCh38 to a median depth of 30× with PEMapper^23^. Genetic variants were jointly called across samples with PECaller (v.2.0.1)^23^ using default parameters.

#### Quality control of genetic data

We prioritized use of WGS over genotyping where available and performed quality control of WGS and genotyping data separately using PLINK^24^. Participants with overall genotyping missingness >10% and sex mismatch were excluded. Variants with evidence of deviation from Hardy–Weinberg equilibrium (*P* < 10^−7^) and missing genotype rate >5% were removed. Genotype data in GRCh37 were converted to GRCh38. We followed the quality-control steps provided by the TOPMed pipeline^25^ for genetic data before imputation. Imputation was performed using the TOPMed imputation panel and server with default parameters^25^. SNPs with imputation quality *R*^2^ > 0.3 were retained.

For WGS, we assessed the coverage, missingness, ratio between the number of transition mutations and the number of transversion mutations, and the silent/replacement ratio (the ratio between synonymous and nonsynonymous mutations). We merged the imputed genotyping data and WGS data and performed further quality control in each population (AA, Hispanic and NHW) separately by excluding variants with any of the following criteria: genotype missing rate >5%, MAF < 5%, nonbiallelic variants and Hardy–Weinberg equilibrium *P* < 10^−7^.

Related individuals were identified using KING (v.2.2.2)^26^, and one in each pair of individuals who were second-degree or closer relatives was randomly removed. Individuals who were population outliers were identified and removed using EIGENSTRAT from EIGENSOFT (v.8.0.0)^27^.

#### Benchmarking ancestry and/or ethnicity

All participants with individual-level genetic data were divided into three populations based on self-report: AA, Hispanic and NHW. Within each self-report population, we removed outliers based on genetic ancestry by performing multidimensional scaling analysis using individual-level genetic data and phase 3 1000 Genomes data as in ref. ^28^. Self-defined Hispanic study participants were combined with the MXL (Mexican ancestry in Los Angeles, CA), PUR (Puerto Rican), CLM (Columbian) and PEL (Peruvian) reference panel populations. Self-defined NHW study participants were combined with the CEU (Utah residents with northern and western European ancestry), TSI (Toscani in Italy), IBS (Iberian population in Spain) and FIN (Finnish) reference panel populations. Self-reported AA participants were combined with the ASW (African ancestry in southwest USA) population. Within each of the three populations, SNPs were filtered to meet the criteria MAF > 5%, data missingness <0.1 and Hardy–Weinberg equilibrium *P* < 10^−7^ using an independent pairwise linkage filter window of 50 kb at 5 kb steps and an *r*^2^ threshold of 0.15. We removed AT, TA, GC and CG markers. Then, for each group, multidimensional scaling analysis was performed using PLINK (v.1.90b53)^24^, and results were visualized along principal components 1 and 2 using R. For each population, samples that were six or more standard deviations from the mean of any reference panel populations were considered outliers and removed (42 in the NHW population, 4 in the AA population and 4 in the Hispanic population).

#### LD panel for each population for PWASs and PMR-Egger

We generated an LD panel for integration of GWASs and brain proteomic and genetic data for each population separately. For NHW, we used the LD reference panel from the 1000 Genomes European data provided in the FUSION pipeline^3^. For African ancestry, we constructed an LD panel using the AFR HapMap SNP sites in the AFR 1000 Genome data^28^. Likewise, for the Hispanic population, we constructed an LD panel using the AMR HapMap SNPs in the AMR 1000 Genome data^28^.

### Proteomic data

#### Proteomic sequencing and database search

Proteomic data were generated by collaborative efforts of the Accelerating Medicines Partnership: Alzheimer’s Disease (AMP-AD)^29^ and AMP-AD Diversity^22,30^ involving multiple research sites. Brain samples were collected by Rush Alzheimer’s Disease Center (*n* = 815), the Mayo Clinic (*n* = 399), Mount Sinai University Hospital (*n* = 205), Emory University (*n* = 129) and the Brain and Body Donation Program at Banner Sun Health (*n* = 200) for proteomic studies as previously reported^7,21,22,30^. All donors or their next of kin provided informed consent, and the study was approved by the respective institutional review boards at all sites. AMP-AD generated data from samples collected by Rush and Banner Sun Health. AMP-AD-Diversity generated data from samples provided by Rush, the Mayo Clinic, Mount Sinai and Emory. Proteomes were profiled using tandem mass tag mass spectrometry as described in detail previously^21,30^. Briefly, postmortem brain samples were homogenized, and proteins were digested with trypsin. Samples were randomized for sex, age, diagnosis, and race and/or ethnicity and labeled with isobaric tandem mass tag peptides. All proteomic sequencing batches included at least one global internal standard (GIS). The GIS were created by aliquoting equal amounts of protein from each sample within a batch; these standards were then digested in parallel with other samples within the batch^31^. Next, high-pH fractionation was performed, and the resulting samples were analyzed by liquid chromatography coupled to tandem mass spectrometry. Raw files were searched using Fragpipe (v.19.0)^32^, MSFragger^32^ (v.3.5) and human proteome database Swiss-Prot^33^ containing 20,402 sequences (downloaded 11 February 2019). Subsequently, we used Post-MSFragger (v.3.6) and Percolator^34^ (v.3.0.5) for peptide–spectrum match validation and Philosopher (v.4.6.0) for protein inference using ProteinProphet (v.4.6.0) and filtering using FDR. The database search yielded a total of 11,748 protein groups from the dorsolateral prefrontal cortex.

#### Quality control and normalization of proteomic data

We examined proteomic assay precision via coefficient of variation (CV) analysis, using the 7 batches with ≥2 GIS (a total of 15 within-batch GIS). Lower CVs reflect lower measurement errors and higher precision. Based on protein abundance before normalization, we calculated the CV for each gene within each batch using the formula CV = s.d.(*x*)/mean(*x*), where *x* is the protein abundance for each gene in the GIS samples within each batch. We found the CVs to have a median of 2.2, interquartile range of 1.8 and range of [0.23–23.1] among 7,675 proteins without any missing values, suggesting very high reproducibility. We performed quality control of proteomic data in the AMP-AD-Rush (*n* = 619), AMP-AD-Banner (*n* = 198) and AMP-AD-Diversity (*n* = 1,105; Rush, Mayo, Mount Sinai, Emory; Supplementary Table 25) datasets separately following our prior approach^7,21,35,36^. Specifically, in each of the three datasets, we performed the following steps. First, we removed duplicate samples (only AMP-AD-Diversity had duplicate samples, and one of the pair was removed). Second, we removed proteins with missing values in more than 50% of the samples. Third, the protein abundance for each gene per sample was normalized using the total abundance of all the proteins in that sample to account for protein loading differences and then log_2_-transformed. Fourth, we performed iterative principal component analysis to remove sample outliers that were more than four standard deviations from the mean of either the first or the second principal component. Fifth, we performed linear regression to estimate and remove the effects of protein sequencing batch, PMI, age at death, sex and clinical diagnosis of cognitive status from the proteomic profiles. In the AMP-AD-Diversity dataset, a subset of the samples did not have PMI (*n* = 149). Thus, we performed regression to remove unwanted technical and biological effects in the subset with PMI separately from the subset without PMI. Last, to enable comparisons across the three datasets (AMP-AD-Rush, AMP-AD-Banner and AMP-AD-Diversity), a *Z*-score transformation of the protein abundance for each protein to a mean of 0 and standard deviation of 1 was applied within each dataset. For proteins with multiple isoforms, we selected the most abundant isoform for investigation. Variance partition plots showing the contributions of technical and biological effects to the proteomic profiles before and after quality control and normalization for these three datasets are provided in Supplementary Figs. 1–3.

Next, we combined the proteomic profiles from the three datasets and retained samples having both proteomic and genetic data. Then we performed additional quality control of the proteomic data in each population (AA, Hispanic and NHW) separately as follows. First, we retained proteins with nonmissing data in at least 50 individuals. Second, we performed surrogate variable analysis (SVA v.3.20.0)^37^ on the proteomic profile to assess potentially hidden confounding variables and regressed out the significant surrogate variables from the normalized proteomic profile. Last, *Z*-score transformation of the surrogate-variable-adjusted protein abundance for each protein was applied to the proteomic profile in each population. After quality control of genetic and proteomic data as described above, a total of 1,362 individuals remained for pQTL mapping. They comprised 181 AA (9,304 proteins), 168 Hispanic (9,036 proteins) and 1,013 NHW (9,725 proteins) individuals (Supplementary Table 24).

### Population-stratified pQTL mapping

pQTL mapping in each population was performed for SNPs present at MAF ≥ 0.05 in a population. We defined *cis* as being within 500 kb up or downstream of the gene. We fitted an LMM with SNP as the independent variable and normalized protein expression as the outcome, adjusting for sex using GEMMA (v.0.98.1)^4^. We chose an LMM as it could account for sample relatedness and population substructure. We calculated the *P* value using the Wald test in GEMMA. As described above, we regressed our the surrogate variables from the proteomic profile of each population before performing pQTL mapping. The genomic inflation factor (*λ*) was calculated by dividing the median of the resulting chi-squared test statistics by the expected median of the chi-squared distribution^38^.

#### Influence of allele frequency differences on population-specific pQTLs

We investigated whether differences in pQTL detection were related to differences in allele frequencies between populations. Here we defined AA-only candidate pQTLs as those identified as pQTLs in AA that were not pQTLs in NHW and were found to have ≤1 minor allele in European populations in the 1000 Genomes WGS data. Among the 144,654 candidate pQTLs in AA from the population-stratified pQTL mapping, we found 14,239 AA-only candidate pQTLs, corresponding to 1,578 independent AA-only candidate pQTLs (after clumping at *r*^2^ < 0.1) and constituting 12.8% of the 7,852 independent candidate pQTLs identified in the AA population. Likewise, we defined NHW-only pQTLs as those that were candidate pQTLs in NHW but not in AA and had ≤1 minor allele in African populations in the 1000 Genomes data. Among the 1,328,595 candidate pQTLs in NHW from the population-stratified pQTL mapping, we found 3,939 NHW-only candidate pQTLs, which corresponded to 1,271 independent NHW-only candidate pQTLs (after clumping at *r*^2^ < 0.1) and constituted 1.1% of the 40,087 independent candidate pQTLs identified in the NHW population.

### Fine-mapping

Multiancestry pQTL fine-mapping was performed with MESuSiE (v.1.0)^8^, which considers only proteins and SNPs that are present in all examined populations. We considered SNPs present at MAF > 0.05 in each population. Inputs to MESuSiE included the summary statistics of the population-stratified pQTL mapping and LD matrix for each population. The LD matrix for each population was generated using the same individual-level genotyping and/or WGS data used in the population-stratified pQTL mapping to ensure there was no mismatch between the population-stratified pQTL summary statistics and the LD matrix. We performed an LD mismatch check following MESuSiE guidelines. We set the *L* parameter (that is, the maximum number of possible credible sets) to the default value of 10. As we performed multiancestry pQTL fine-mapping in three populations, there were seven possible pQTL categories: (1) shared across AA, Hispanic and NHW; (2) shared between AA and Hispanic; (3) shared between AA and NHW; (4) shared between Hispanic and NHW; (5) specific to AA; (6) specific to Hispanic; and (7) specific to NHW. The output from MESuSiE included the PIP for each of these categories, referred to as the category.PIP. We then applied additional filters to the MESuSiE results to remove causal pQTLs with category assignments discordant with the population-specific pQTL results. Specifically, among causal pQTLs categorized as shared across populations, we filtered out those that were not nominally significant in each population (that is, *P* > 0.05) or had discordant beta values. Likewise, for the causal pQTLs categorized as population-specific, we filtered out those that had population-stratified pQTL *P* < 0.05 in other populations.

#### pQTL fine-mapping in NHW with SuSiE

To compare the size of the 95% credible sets between multiancestry pQTL fine-mapping and NHW-only pQTL fine-mapping, we performed pQTL fine-mapping in NHW with susieR (v.0.12.35)^9^ following its default pipeline. SuSiE was designed to identify as many credible sets as the data would support, each with as few variants as possible. For a given gene and its corresponding variants in the *cis*-regulatory region, the output was the number of credible sets that had 95% probability of containing a variant with nonzero causal effect. We set the maximum number of credible sets for a gene to be ten (the default value).

#### Influence of sample size on population-shared and -specific pQTLs

We downsampled the NHW proteomic dataset from 1,013 to 191 to make it comparable to the sample sizes for the AA and Hispanic populations. The smaller NHW group (EUR-2) had similar distributions of age, sex and PMI as the larger NHW group. We performed population-stratified pQTL mapping for each group (EUR-2 *n* = 191; AA *n* = 181; Hispanic *n* = 168) and used the pQTL summary statistics as an input for multiancestry pQTL fine-mapping with MESuSiE.

### Genomic-site-type enrichment

Genomic-site-type annotations for the 3,643,245 SNPs tested in all three populations in the population-stratified pQTL analyses were obtained using Bystro (v.2.0.0-beta1)^39^. SNPs were annotated with the following site types: nonsynonymous, synonymous, UTR or intronic. In each case, the annotation was assigned to a SNP if and only if it pertained to one or more of the genes tested for the SNP. Promoter overlap for each SNP was determined using the promoter-like candidate *cis*-regulatory elements from ENCODE^40^. Fisher’s exact test was used to test enrichment of each site type in two sets of pQTLs: the multiancestry causal pQTLs from MESuSiE and the shared candidate pQTLs from the population-stratified pQTL analyses. As the shared candidate pQTLs included many SNPs in LD, a set of approximately independent SNPs was selected by applying the --clump function from PLINK to greedily select pQTLs with pairwise LD < 0.1. For both sets, a SNP was counted as ‘in the set’ if it was a pQTL for any gene.

### Cross-ancestry prediction of genetically regulated protein abundance

Focusing on the 858 multi-ancestry causal pQTLs, we included SNPs in their corresponding 95% credible sets. Then, we computed the effect of these SNPs on protein abundance (also referred to as protein ‘weights’) using NHW genetic and proteomic data and multiple predictive models (top1, blup, lasso, enet and bslmm) following the FUSION framework^3^. We extracted the protein weights from the most predictive models. Next, we estimated the genetically regulated protein abundance for the pGenes corresponding to the multiancestry causal pQTLs using AA genotyping and the above protein weights. Subsequently, we calculated the *R*^2^ and *P* value for regression of the predicted AA proteomic expression on the measured AA proteomic expression to provide estimates of cross-ancestry prediction accuracy.

In addition, we performed same-ancestry prediction following the pipeline described above using AA genetic and proteomic data for the pGenes corresponding to the 858 multiancestry causal pQTLs. Following the FUSION framework, we used all the SNPs corresponding to the pGenes for the 858 multiancestry causal pQTLs. The output provided cross-validation *R*^2^ values for the best-performing models. The *R*^2^ metric reflects the percentage variance of protein abundance explained by the genetically regulated protein level, with higher values corresponding to more variance explained. To compare the cross-ancestry to the same-ancestry prediction, we took the ratio of the cross-ancestry and same-ancestry cross-validation *R*^2^ values.

### *π*_1_ replication rate for pQTLs

To determine the *π*_1_ rate for the multiancestry causal pQTLs in each of the populations, we first estimated *π*_0_, which is the proportion of true null hypotheses among a set of tests, using R package qvalue (v.2.15.0). In the case of our application to *P* values from the LMM tests, the null hypothesis was that the SNP had zero effect on protein expression. *π*_1_, the proportion of true alternative hypotheses, was defined as 1 − ${\pi }_{0}$.

To compare the pQTLs and pGenes identified here in the NHW population with those from our previous published brain pQTL study using a smaller dorsolateral prefrontal cortex dataset^7^, we extracted significant pQTLs and pGenes from each study (FDR < 0.05) and identified overlapping and unique findings. To estimate *π*_1_ rates using the current brain pQTLs as the discovery set and published plasma pQTLs as replication sets, we extracted the population-stratified brain pQTLs (at FDR < 0.05) in the current study and examined them in each of the published plasma datasets to estimate *π*_0_. In addition, we estimated *π*_1_ for the multiancestry causal pQTLs (*n* = 858) in each of the two plasma pQTL datasets in African and European ancestry populations separately following a similar procedure.

To determine which pGenes were shared between brain and plasma, we focused on European ancestry owing to the higher sample sizes and statistical power. We defined pGenes as proteins with *cis*-pQTLs at FDR < 5%. To make the plasma pGene definition comparable to our current brain pGene definition, for each protein, we considered SNPs within 500 kb of the gene and estimated the FDR using published plasma pQTL summary statistics in each of the European plasma datasets (Icelanders^41^, ARIC^5^ and UKB^6^). For each comparison, we considered only genes available in both brain and plasma datasets. We examined the overlap between brain and plasma pGenes (‘shared’ pGenes) and the pGenes found only in brain analysis or plasma analysis (‘unique’ pGenes) for each plasma pQTL dataset (Supplementary Tables 25–27). Of our brain pGenes, we found that 91% shared pGenes with plasma pGenes from the Icelanders dataset, 84% shared pGenes with ARIC plasma pGenes and 98% shared pGenes with UKB plasma pGenes (Supplementary Table 28).

### GWAS summary statistics

We had access to GWAS summary statistics for 15 psychiatric traits: alcohol use disorder^42^, anorexia nervosa^43^, anxiety^44^, ADHD^45^, autism spectrum disorder^46^, bipolar disorder^47^, cannabis use disorder^48^, insomnia^49^, major depressive disorder^50,51^, neuroticism^52^, opioid addiction^53^, PTSD^54^, schizophrenia^55^, suicide attempt^56^ and tobacco use^57^. We also had summary statistics for six neurologic traits: Alzheimer’s disease^58,59^, amyotrophic lateral sclerosis^60^, frontotemporal dementia^61^, Lewy body dementia^62^, Parkinson’s disease^63,64^ and stroke^65^. We used these results to integrate brain proteomes with GWASs in PWASs, SMR and PMR-Egger as described below.

### Integrating GWASs with brain proteomes using PWASs and SMR

For each of the 21 psychiatric and neurologic conditions, we integrated GWAS summary statistics with brain proteomic and genetic data in each population separately using the population-matching GWAS, brain proteome and LD reference panel. All gene coordinates were based on GRCh38. We performed two independent but complementary integration approaches. First, we performed a PWAS of each trait using FUSION^3^ (https://github.com/gusevlab/fusion_twas, commit e1ba5f7). We restricted the genotype data to the SNPs in the LD reference panel in each population. SNP-based heritability for each protein was estimated, and proteins with SNP-based heritability *P* < 0.01 were considered to be heritable. For each heritable protein, we estimated the effect of a set of SNPs within a 500-kb window of the gene on its protein abundance. We applied BLUP, LASSO, elastic net and BSLMM prediction models and kept the weights from the best-performing prediction model. Subsequently, we integrated the brain protein weights with each of the GWAS summary statistics to perform a PWAS for each trait in each population for which GWAS results were available, to identify the *cis*-regulated proteins associated with the trait. We defined significant proteins as those with FDR < 0.05.

In the second approach, among the significant *cis*-regulated proteins identified in the PWAS described above, we performed summary data-based Mendelian randomization with SMR^10^ to test whether the brain protein mediated the association between the SNP and trait. We used the population-stratified pQTLs and GWAS summary statistics for each trait in each population separately. As mediation can arise from causality, pleiotropy or LD, we then used HEIDI^10^ to test for and remove associations likely to be due to LD (that is, HEIDI *P* < 0.05). We defined proteins consistent with a causal role as those with PWAS FDR < 0.05, SMR *P* < 0.05 and HEIDI *P* > 0.05 and a consistent direction of association between protein and trait in the PWAS and SMR.

### PMR-Egger using population-stratified data

As an alternative approach to the above PWAS and SMR framework, we used PMR-Egger as implemented in PMR (https://github.com/yuanzhongshang/PMR, commit 7e49f14) to perform SNP–protein–disease causal inference using pQTL and GWAS summary statistics. PMR-Egger is a probabilistic two-sample Mendelian randomization that also controls for horizontal pleiotropy^11^. Unlike traditional Mendelian randomization approaches that use independent instruments, PMR uses correlated instruments and has similar detection power to PWAS approaches such as FUSION^3^. It also accounts for horizontal pleiotropy and provides a *P* value for the effect of horizontal pleiotropy. We ran PMR with function PMR_summary_Egger for each gene in each population (NHW, AA and Hispanic) with our pQTL data and published GWAS results, the same data used in the PWAS and SMR analyses described above, following the instructions in the developer’s example (https://github.com/yuanzhongshang/PMR/tree/master/example). We ran PMR-Egger only for genes that passed the heritability test in the PWAS analysis and calculated an LD matrix for each population using individual-level genetic data from postmortem brain donors. To account for potential mismatches between GWAS data and the LD matrix, we performed an LD mismatch check per MESuSiE guidelines and removed mismatching SNPs before the PMR-Egger run.

### Identifying multiancestry causal pQTLs in psychiatric and neurologic conditions through Mendelian randomization

In each population separately, we modeled the 858 multiancestry causal pQTLs as the instrumental variable in Mendelian randomization analysis with SMR^10^ using the population-matching GWAS and pQTL summary statistics. We declared multiancestry causal pQTLs to be consistent with a causal role if they had SMR FDR < 0.05 and HEIDI *P* > 0.05 for the trait of interest.

### Physical PPIs

The BioGRID database (v.4.4.232; 28 April 2024)^66^ was used to obtain pairwise protein interactions containing only human gene symbols, which were further filtered to include only physical PPIs such as physical associations and direct interactions, associations and colocalization.

### Gene set enrichment analysis

For causal proteins for each trait, gene set enrichment analysis was performed using GO-Elite (v.1.2.5)^67^ with the background gene set of 9,725 proteins that remained after comprehensive quality control. Causal proteins were subjected to Fisher’s exact overlap test and *Z*-test in the Python command-line version of GO-Elite, setting the species as *Homo sapiens* and using the current annotation databases for gene ontology biological processes, molecular functions, cellular components, Wiki pathways, KEGG, REACTOME and CORUM (downloaded April 2024)^68^.

### Reporting summary

Further information on research design is available in the Nature Portfolio Reporting Summary linked to this article.

## Online content

Any methods, additional references, Nature Portfolio reporting summaries, source data, extended data, supplementary information, acknowledgements, peer review information; details of author contributions and competing interests; and statements of data and code availability are available at 10.1038/s41588-025-02291-2.

## Supplementary information

Supplementary Information Supplementary Notes A–C and Figs. 1–3.

Reporting Summary

Supplementary Tables Supplementary Tables 1–28.

## Source data

Source Data Extended Data Fig. 1 Underlying data for Extended Data Fig. 1.

## Data Availability

The data and results are available via Synapse at 10.7303/syn64600176. These data include links to the raw and processed proteomic data, pQTL results and protein weights from FUSION. These data and results are in whole or in part based on data obtained from the AMP-AD Knowledge Portal (https://adknowledgeportal.synapse.org/Explore/Programs/DetailsPage?Program=AMP-AD), a platform for access to data, analyses and tools generated by the AMP-AD Target Discovery Program and other programs supported by the National Institute on Aging to enable open-science practices and accelerate translational learning. The data, analyses and tools are shared early in the research cycle without a publication embargo on secondary use. Data are available for general research use according to the requirements for data access and data attribution described at https://adknowledgeportal.org/DataAccess/Instructions. Source data are provided with this paper.

## Code availability

Our code is available via Zenodo at 10.5281/zenodo.15826233 (multiancestry brain pQTL)^69^ and 10.5281/zenodo.15826249 (PE Caller)^70^.
