# Supplementary material for: Multiancestry brain pQTL fine-mapping and integration with genome-wide association studies of 21 neurologic and psychiatric conditions
Source: Nat Genet. 2025 Sep 8;57(9):2156–65. doi: 10.1038/s41588-025-02291-2 (PMC12425806; doi:10.1038/s41588-025-02291-2)

nature genetics

Supplementary information https://doi.org/10.1038/s41588-025-02291-2

Multiancestry brain pQTL fine-mapping and integration with genome-wide association studies of 21 neurologic and psychiatric conditions

In the format provided by the authors and unedited

SUPPLEMENTARY NOTE

A. Comparing current brain pQTLs with published brain pQTL and plasma pQTL studies

We compared the current NHW brain pQTL findings (in n=1013 NHW brain proteomes) with the largest published brain pQTL study in European ancestry (n=716)[1], of which 674 donors were common between the two datasets. We found that 49% of the brain pQTLs and 28% of the pGenes identified here are novel, likely due to the larger brain proteome sample size. Among the candidate pQTLs found in both the current and published studies, the $\pi_1$replication rate was 0.94, suggesting a high level of concordance (**Table A**).

Regarding plasma pQTLs, we found three large published studies on plasma pQTLs, of which one profiled plasma proteomes in Icelandic participants of EUR ancestry using SomaScan (Ferkingstad et al, n=35,559)[2] and two examined plasma pQTLs in multiple ancestries – i) Zhang et al used SomaScan to profile proteins in ARIC participants (AFR n=1,871; EUR n=7,213)[3] and ii) Sun and colleagues used Olink to profile proteins in UKB participants (AFR n=931, EUR n=34,557)[4]. Both studies provided plasma pQTLs in different ancestries without explicitly examining whether they are shared between ancestries or specific to an ancestry. We aimed to determine the concordance between our ancestry-stratified brain pQTLs (discovery set, n=1013 EUR and 181 AFR) and the published ancestry-stratified plasma pQTLs (replication set) using $\pi_1$replication statistics and concordance of direction of association. In EUR ancestry, π_1 was 0.53 for Ferkingstad et al (beta concordance: 0.75; n=35,559, SomaScan), 0.47 for Zhang et al (beta concordance: 0.77; n=7213, SomaScan), and 0.72 for Sun et al (beta concordance: 0.74; n=34,557; Olink; **Table A**). In AFR ancestry, π_1 was 0.51 for Zhang et al (beta concordance: 0.86; n=1871, SomaScan) and 0.63 for Sun et al (beta concordance: 0.84; n=931, Olink; **Table A**).

Next, we sought to determine the percentage of the brain multi-ancestry causal pQTLs that are also plasma pQTLs in AFR or EUR ancestry using the published SomaScan and Olink-based plasma pQTLs, respectively. We found moderate replication rates in either ancestry in plasma pQTLs (SomaScan plasma pQTLs AFR π_1 = 0.34, EUR π_1 = 0.57; Olink plasma pQTLs: AFR π_1 = 0.47, EUR π_1 = 0.77, **Table B**). As a frame of reference, Sun et al found the percentage of replicated plasma pQTLs to be 0.84 for same tissue and platform and 0.38 for same tissue and different platform[4]. In that light, our replication rates are relatively high considering we are comparing across different tissues and platforms, lending support to our findings of putatively shared causal pQTLs.

Table A: π1 rate for current brain proteomic data as the discovery set (EUR=1013; AFR=181) and the published studies as the replication sets

Replication set	**Brain pQTL**<br>Mass spectrometry<br>Wingo et al<br>**EUR**<br>(n=716)	**Plasma pQTL - UKB**<br>Olink<br>Sun et al<br>**AFR**<br>(n=931)	 <br><br>**EUR**<br>(n=34,557)	**Plasma pQTL - ARIC**<br>SomaScan<br>Zhang et al<br>**AFR**<br>(n=1871)	 <br><br>**EUR**<br>(n=7213)	**Plasma pQTL - Iceland**<br>SomaScan<br>Ferkingstad et al<br>**EUR**<br>(n=35,559)
**π_1 rate**	0.94	0.63	0.72	0.51	0.47	0.53
**beta concordance** (brain-brain or brain-blood*)	0.94	0.84	0.74	0.86	0.77	0.75

*only in SNPs that are pQTLs in both brain and blood at FDR<0.05

Table B: π_1 rate for brain multi-ancestry causal pQTLs (Discovery) in plasma pQTLs (Replication)

	**Plasma pQTL in UKB (Olink)**		**Plasma pQTL in ARIC (SomaScan)**	
	**AFR** (n=931)	**EUR** (n=34,557)	**AFR** (n=1871)	**EUR** (n=7213)
**π_1 rate**	0.47	0.77	0.34	0.59
**beta concordance** (brain-blood)*	0.83	0.83	0.80	0.85

In addition, we examined the overlap between brain and plasma pGenes (referred to as 'shared' pGenes) and the pGenes found only in brain analysis or plasma analysis ('unique' pGenes) for each plasma

pQTL dataset (Supplementary Tables 26-28). Between our brain pGenes and plasma pGenes, we found 91% shared pGenes with Icelanders plasma pGenes, 84% shared with ARIC plasma pGenes, and 98% shared with UKB plasma pGenes (Supplementary Table 29).

We next assessed whether protein abundance or coefficient of variation (CV) might contribute to the sharing of pGenes between brain and plasma. To do that, we compared the median protein abundance and CV, respectively, between shared pGenes and unique pGenes within each brain-plasma dataset pair using the two-sided Wilcoxon rank-sum test. In the brain-ARIC dataset, shared pGenes exhibited significantly higher brain protein abundance ($p = 0.027$) and lower brain CVs ($p = 0.025$) compared to pGenes unique to either tissue. However, in the two larger plasma datasets, we found no significant differences in brain protein abundance or CV between shared and unique pGenes in the brain-Icelandic or brain-UK Biobank (UKB) dataset pair.

B. Integrating GWAS with human brain proteogenomic data using PMR-Egger

As a secondary analysis, we performed integration of population-matched brain proteomic with GWAS results in each ancestry/ethnicity separately for these 21 neurologic and psychiatric conditions using PMR-Egger, which is a probabilistic Mendelian randomization (MR) framework that tests and controls for horizontal pleiotropy[5]. By design, PMR-Egger uses correlated SNPs as instrument variables similar to the PWAS approach and thus is analogous to the PWAS/SMR approach above. We declared candidate causal proteins as those with PMR-Egger FDR $p<0.05$ and PMR-Egger-pleiotropy $p>0.05$. We found 695 candidate causal proteins in African ancestry (Supplementary Table 12), 36 in Hispanic (Supplementary Table 13), and 3206 in NHW (Supplementary Table 14), which is many more than what we found through the PWAS/SMR framework (3 causal proteins in African ancestry, 0 in Hispanic, and 1137 in NHW; Supplementary Table 15 and Table 2). Next, we determined the overlap in causal proteins between the two approaches in NHW. An average of 54.5% (median: 60.4%) of the PWAS/SMR causal proteins are also PMR-Egger causal proteins, and 26.8% (median: 25.0%) of PMR-Egger causal proteins are also PWAS/SMR causal proteins (Supplementary Table 16). These findings suggest that while the PWAS/SMR approach is more conservative, it is less prone to false positive findings.

C. From candidate causal proteins to molecular processes and drug repurposing

Candidate causal proteins highlight and expand constituents of disease-relevant pathways. In Alzheimer's disease (AD), 9 candidate causal proteins we identified are involved two key hallmarks of AD pathophysiology: creation and clearance of amyloid-β peptide and microtubule protein tau phosphorylation (**Figure A**). In particular, 6 AD causal proteins – TSPAN14, CD2AP, CTSH, GRN, TMEM106B, and ACE – have been previously reported to play an essential role in processing and degradation of amyloid-β (**Figure A**)[6-12]. Four other AD causal proteins – EGFR, PIK3IP1, PPP2R2A, and PPP1R14B – were reported to participate in tau phosphorylation[13-17] (**Figure A**). Moreover, 3 AD causal proteins – EGFR, DUSP12, MAP2K1 – are part of the biological pathway activated by amyloid-β in releasing pro-inflammatory cytokines[13,18,19] (**Figure B**). Another four AD causal proteins – EGFR, CTSH, GRN, and TMEM106B – participate in phagocytosis of amyloid-β in microglia[10,11,20-22] (**Figure B**). For anxiety, 3 of its candidate causal proteins – MAPT, STX1B, and BSN – have been reported to play essential roles in neurotransmission and synaptic signaling[23-27] (**Figure C**). These are examples highlighting proteins with genetic evidence driving disease pathophysiology.

Figure A Schematic diagram of APP processing and EGFR signaling in neurons and links to causal proteins in Alzheimer's disease

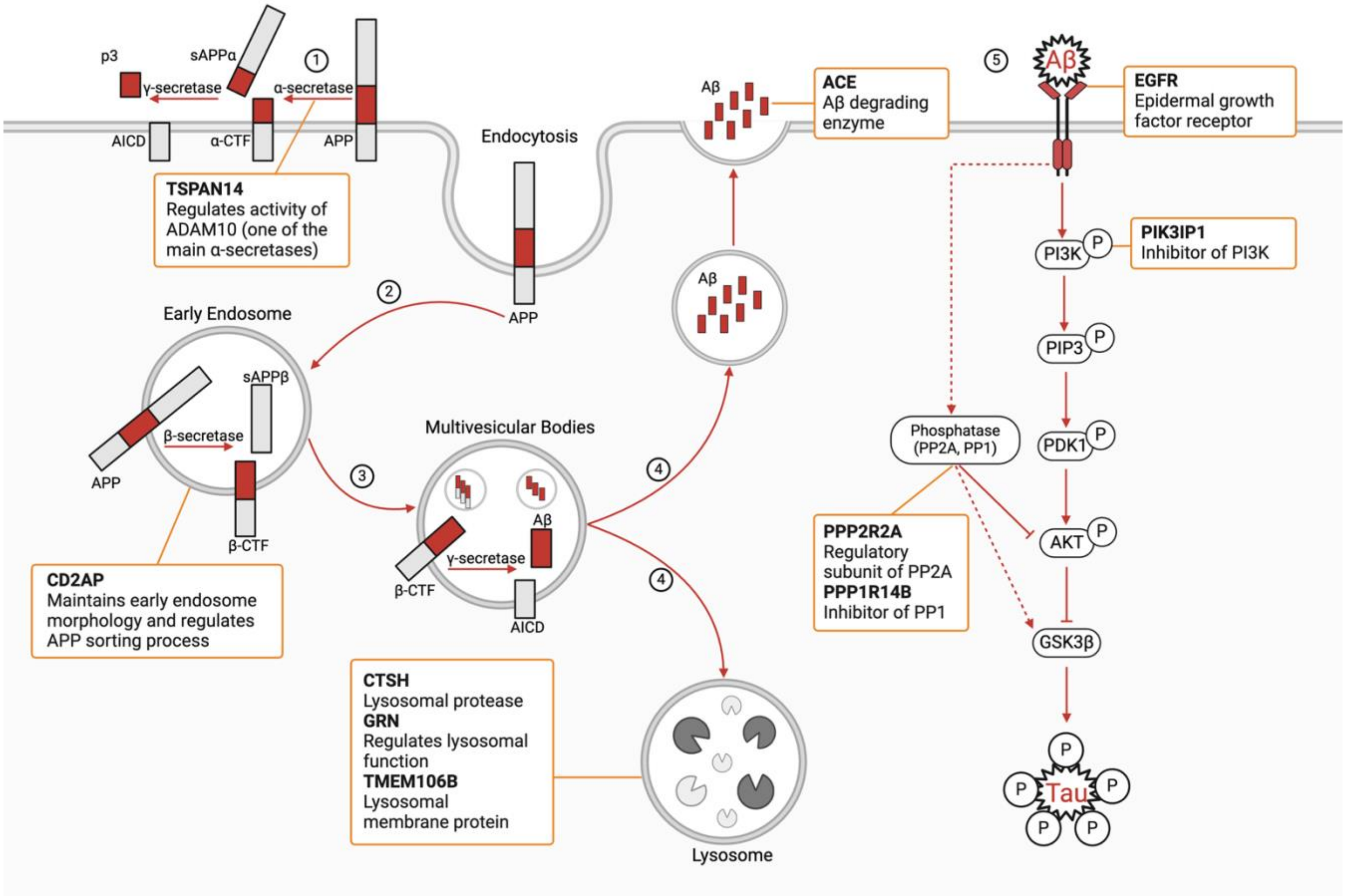

[1] In the non-amyloidogenic pathway, APP is cleaved by α-secretase and γ-secretase at the plasma membrane, generating soluble APPα (sAPPα), APP intracellular domain (AICD), and p3 fragments[6]. Tspan14 regulates the trafficking and function of the ADAM10[7,8]. [2] In the amyloidogenic pathway, full length APP is internalized into early endosomes, where β-secretase generates soluble APPβ (sAPPβ) and β-secretase C-terminal fragment (β-CTF)[6]. CD2AP influences APP sorting process in early endosomes[9]. [3] The β-CTF fragment is trafficked to the multivesicular bodies (MVBs), where γ-secretase cleaves it to produce β-amyloid (Aβ) and AICD[6]. [4] Aβ can either be degraded by lysosomes or released into the extracellular space. CTSH[22], GRN[10] and TMEM106B[11] are associated with lysosomal function. ACE is a membrane-bound Aβ degrading enzyme that regulates Aβ levels[12]. [5] Extracellular Aβ can induce sustained activation of EGFR, leading to the activation of phosphatases such as PP1 and PP2A[13]. These phosphatases inhibit Akt and activate GSK3β in the PI3K/Akt signaling pathway, leading to the hyperphosphorylation of tau protein[13,14]. PIK3IP1 is an inhibitor of PI3K[15]. PPP2R2A is a regulatory subunit of PP2A[16], and PPP1R14B is an inhibitor of PP1[17].

Figure B: Schematic diagram of EGFR signaling and Aβ phagocytosis in microglia, both of which involve the causal proteins we identified in Alzheimer's disease.

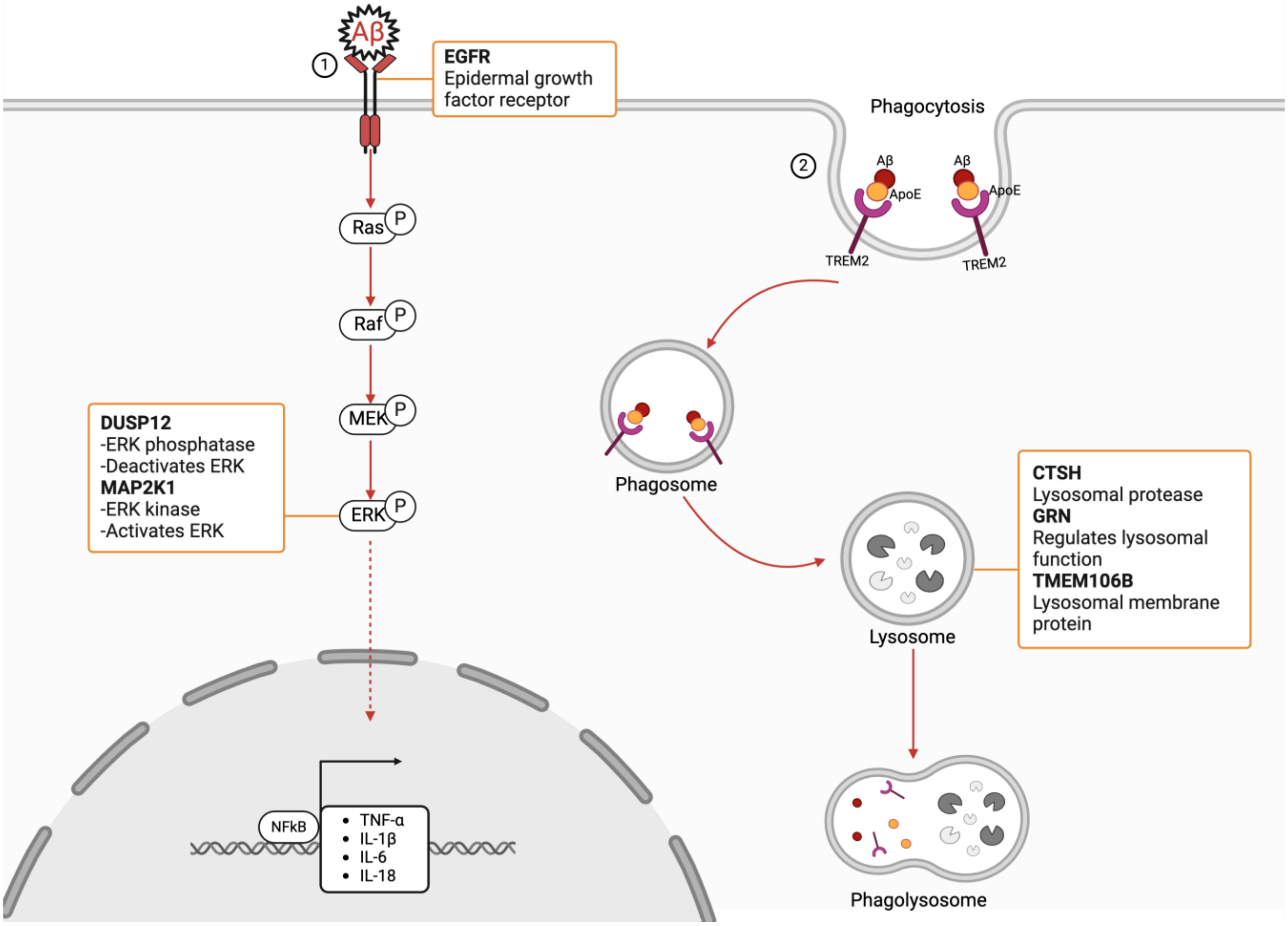

[1] Aβ can induce EGFR and ERK activation in microglial cells, which further activate the nuclear factor-κB (NF-κB) pathway, leading to the expression of pro-inflammatory cytokines such as TNF-α, IL-1β, IL-6, and IL-18[13]. DUSP12 acts as an ERK phosphatase[18], and MAP2K1 functions as an ERK kinase[19]. [2] APOE-chaperoned Aβ can induce TREM2-dependent microglial phagocytosis, which enables microglia to internalize and degrade Aβ within phagolysosomes[20,21]. CTSH[22], GRN[10] and TMEM106B[11] are causal proteins associated with lysosomal function.

Figure C: Schematic diagram of synaptic transmission and links to causal proteins in anxiety

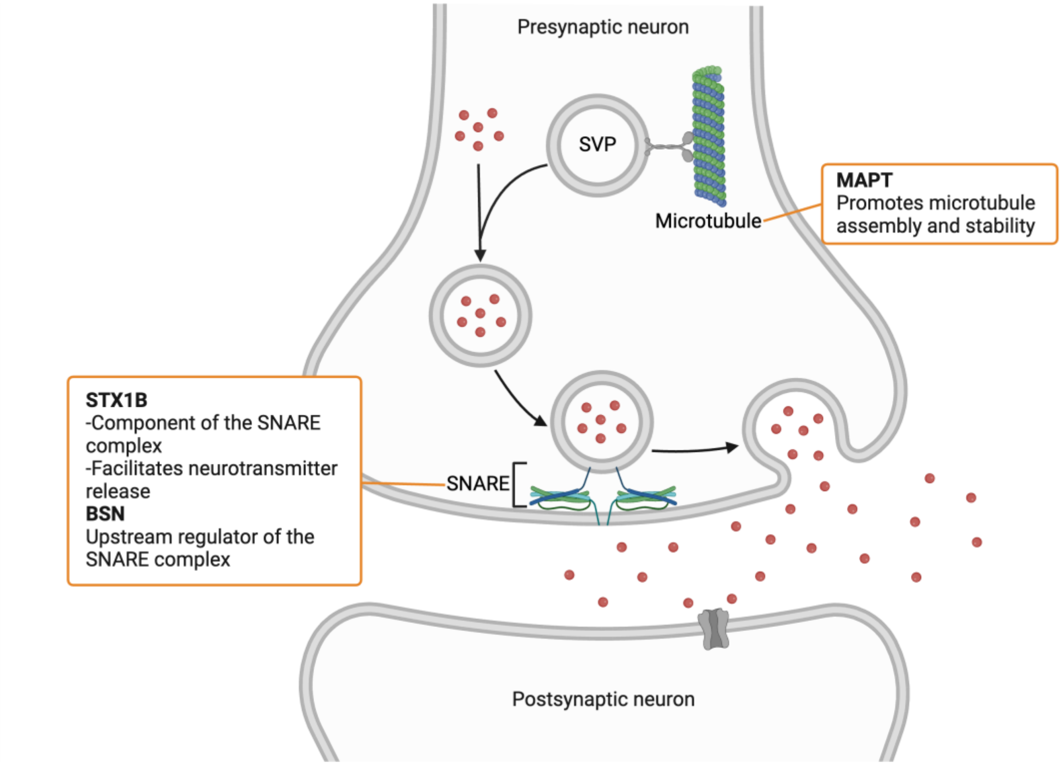

In the presynatic neuron, synaptic vesicle precursors (SVP) are transported to the synapse along the microtubules[23]. Once at the synapse, they are loaded with neurotransmitters and become synaptic vesicles[24]. The synaptic vesicles are then fused with the presynaptic membrane and this process involves the SNARE complex[25]. Among the causal proteins in anxiety, MAPT is a microtubule-associated protein that promotes microtubule assembly and stability[26]. STX1B is a core component of the SNARE complex[25,28], and BSN is a presynaptic scaffold protein that regulates the SNARE complex[27]. Together, the anxiety causal proteins we identified - MAPT, STX1B, BSN - play important roles in neurotransmission and synaptic signaling.

References

1. Wingo, A.P., *et al.* Sex differences in brain protein expression and disease. *Nature medicine* **29**, 2224-2232 (2023).
2. Ferkingstad, E., *et al.* Large-scale integration of the plasma proteome with genetics and disease. *Nature genetics* **53**, 1712-1721 (2021).
3. Zhang, J., *et al.* Plasma proteome analyses in individuals of European and African ancestry identify cis-pQTLs and models for proteome-wide association studies. *Nature genetics* **54**, 593-602 (2022).
4. Sun, B.B., *et al.* Plasma proteomic associations with genetics and health in the UK Biobank. *Nature* **622**, 329-338 (2023).
5. Yuan, Z., *et al.* Testing and controlling for horizontal pleiotropy with probabilistic Mendelian randomization in transcriptome-wide association studies. *Nat Commun* **11**, 3861 (2020).
6. Polanco, J.C., *et al.* Amyloid-beta and tau complexity - towards improved biomarkers and targeted therapies. *Nat Rev Neurol* **14**, 22-39 (2018).
7. Saint-Pol, J., *et al.* Regulation of the trafficking and the function of the metalloprotease ADAM10 by tetraspanins. *Biochem Soc Trans* **45**, 937-944 (2017).
8. Khezri, M.R., Mohebalizadeh, M. & Ghasemnejad-Berenji, M. Therapeutic potential of ADAM10 modulation in Alzheimer's disease: a review of the current evidence. *Cell Commun Signal* **21**, 60 (2023).
9. Tao, Q.Q., Chen, Y.C. & Wu, Z.Y. The role of CD2AP in the Pathogenesis of Alzheimer's Disease. *Aging Dis* **10**, 901-907 (2019).
10. Paushter, D.H., Du, H., Feng, T. & Hu, F. The lysosomal function of progranulin, a guardian against neurodegeneration. *Acta Neuropathol* **136**, 1-17 (2018).
11. Feng, T., Lacrampe, A. & Hu, F. Physiological and pathological functions of TMEM106B: a gene associated with brain aging and multiple brain disorders. *Acta Neuropathol* **141**, 327-339 (2021).
12. Chen, G.F., *et al.* Amyloid beta: structure, biology and structure-based therapeutic development. *Acta Pharmacol Sin* **38**, 1205-1235 (2017).
13. Jayaswamy, P.K., *et al.* Implicative role of epidermal growth factor receptor and its associated signaling partners in the pathogenesis of Alzheimer's disease. *Ageing Res Rev* **83**, 101791 (2023).
14. Beurel, E., Grieco, S.F. & Jope, R.S. Glycogen synthase kinase-3 (GSK3): regulation, actions, and diseases. *Pharmacol Ther* **148**, 114-131 (2015).
15. Jia, Y., *et al.* PIK3IP1: structure, aberration, function, and regulation in diseases. *Eur J Pharmacol* **977**, 176753 (2024).
16. Shi, Y. Serine/threonine phosphatases: mechanism through structure. *Cell* **139**, 468-484 (2009).
17. Tountas, N.A. & Brautigan, D.L. Migration and retraction of endothelial and epithelial cells require PHI-1, a specific protein-phosphatase-1 inhibitor protein. *J Cell Sci* **117**, 5905-5912 (2004).
18. An, N., *et al.* Dual-specificity phosphatases in mental and neurological disorders. *Prog Neurobiol* **198**, 101906 (2021).
19. Roskoski, R., Jr. ERK1/2 MAP kinases: structure, function, and regulation. *Pharmacol Res* **66**, 105-143 (2012).
20. Yeh, F.L., Wang, Y., Tom, I., Gonzalez, L.C. & Sheng, M. TREM2 Binds to Apolipoproteins, Including APOE and CLU/APOJ, and Thereby Facilitates Uptake of Amyloid-Beta by Microglia. *Neuron* **91**, 328-340 (2016).
21. Zhao, Y., *et al.* TREM2 Is a Receptor for beta-Amyloid that Mediates Microglial Function. *Neuron* **97**, 1023-1031 e1027 (2018).
22. Patel, S., Homaei, A., El-Seedi, H.R. & Akhtar, N. Cathepsins: Proteases that are vital for survival but can also be fatal. *Biomed Pharmacother* **105**, 526-532 (2018).
23. Aiken, J. & Holzbaur, E.L.F. Cytoskeletal regulation guides neuronal trafficking to effectively supply the synapse. *Curr Biol* **31**, R633-R650 (2021).
24. Jahn, R. & Fasshauer, D. Molecular machines governing exocytosis of synaptic vesicles. *Nature* **490**, 201-207 (2012).
25. Jahn, R., Cafiso, D.C. & Tamm, L.K. Mechanisms of SNARE proteins in membrane fusion. *Nat Rev Mol Cell Biol* **25**, 101-118 (2024).
26. Derisbourg, M., *et al.* Role of the Tau N-terminal region in microtubule stabilization revealed by new endogenous truncated forms. *Sci Rep* **5**, 9659 (2015).

27. Montenegro-Venegas, C.*, et al.* Bassoon controls synaptic vesicle release via regulation of presynaptic phosphorylation and cAMP. *EMBO Rep* **23**, e53659 (2022).
28. Chen, F.*, et al.* Dysfunction of the SNARE complex in neurological and psychiatric disorders. *Pharmacological research* **165**, 105469 (2021).

Supplementary Figures: Variance partition plots for the technical and biological effects on proteomic profiles before and after quality control and normalization of proteomic data.

Supplementary Figure 1: AMP-AD-Rush proteomic profile

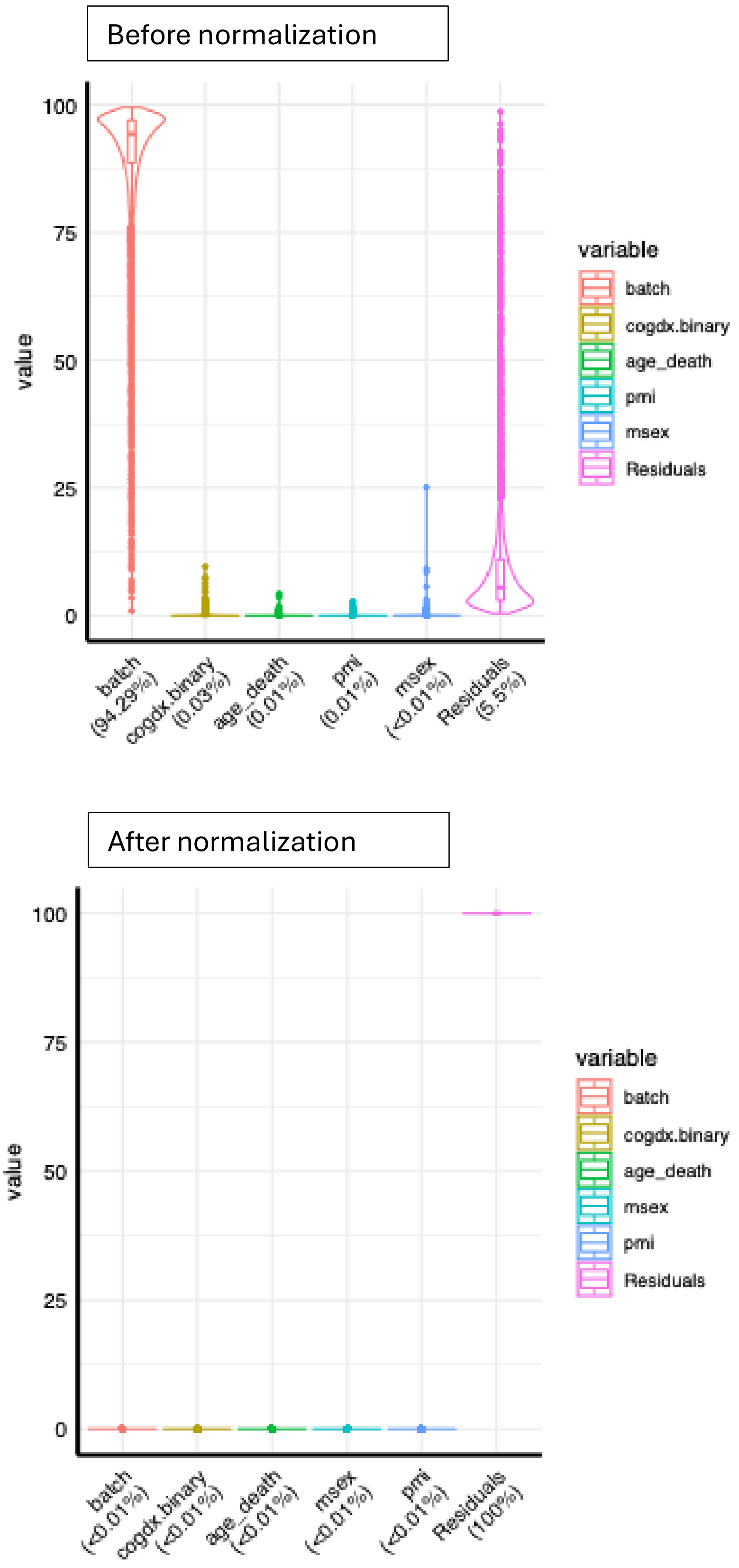

Supplementary Figure 2: AMP-AD Banner proteomic profile

Before normalization

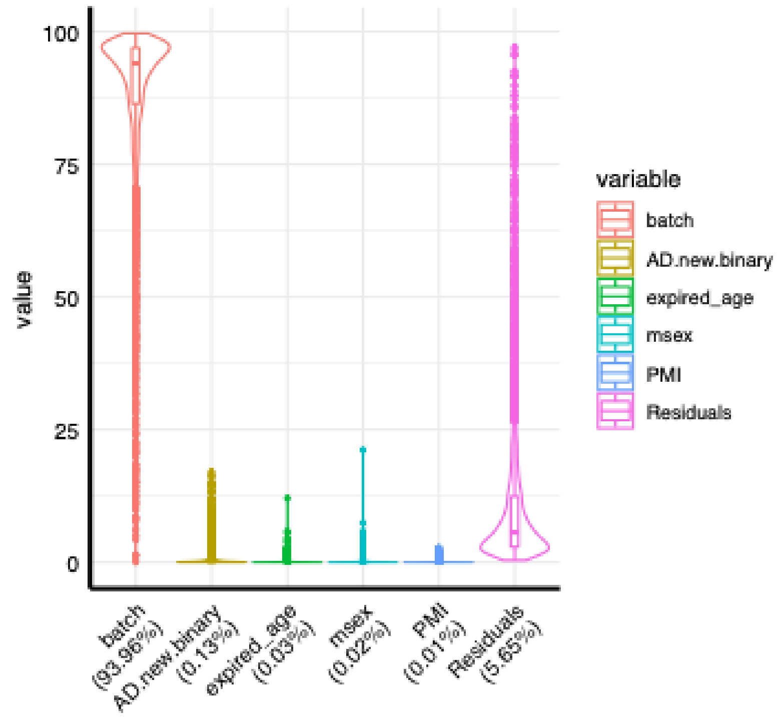

After normalization

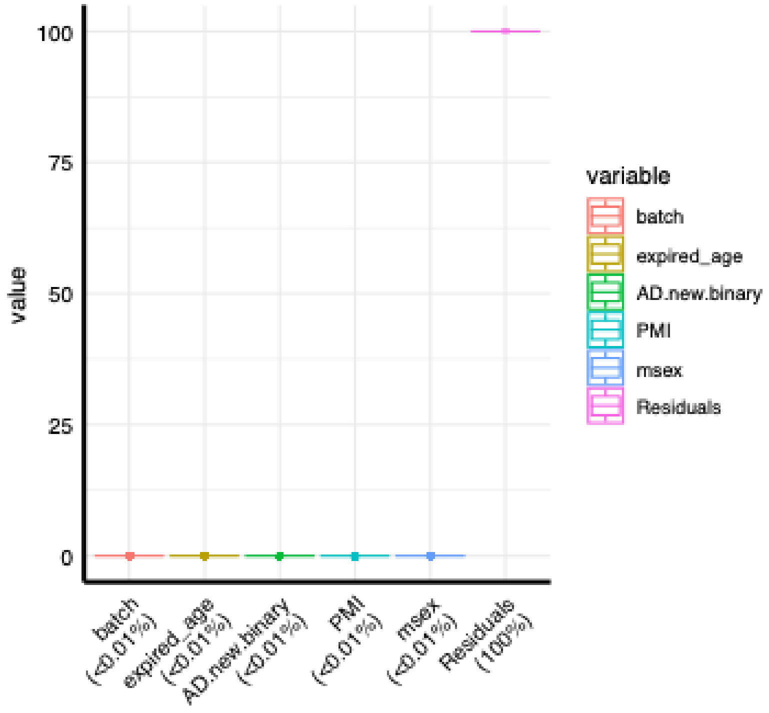

Supplementary Figure 3: AMP-AD-Diversity proteomic profile

Before normalization

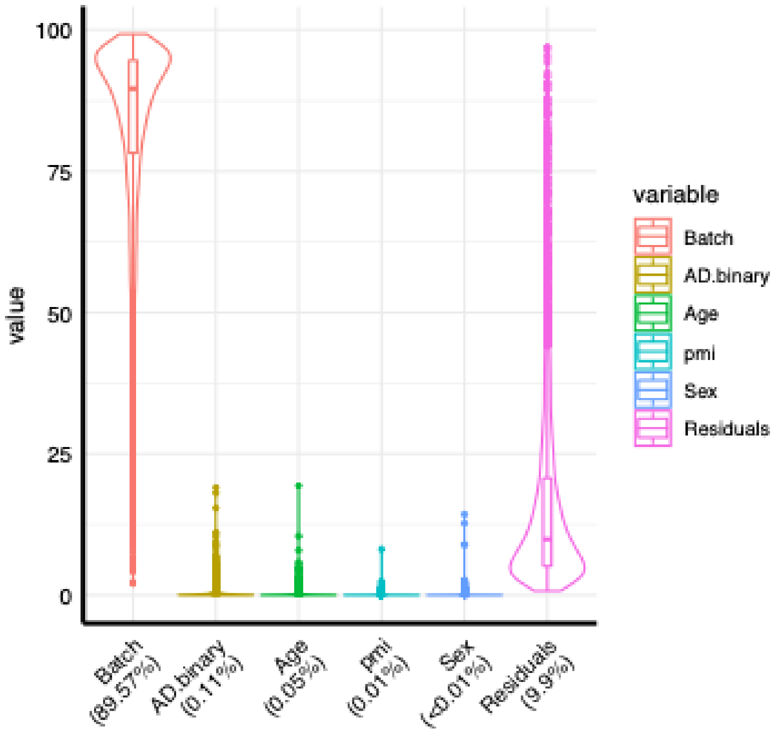

After normalization

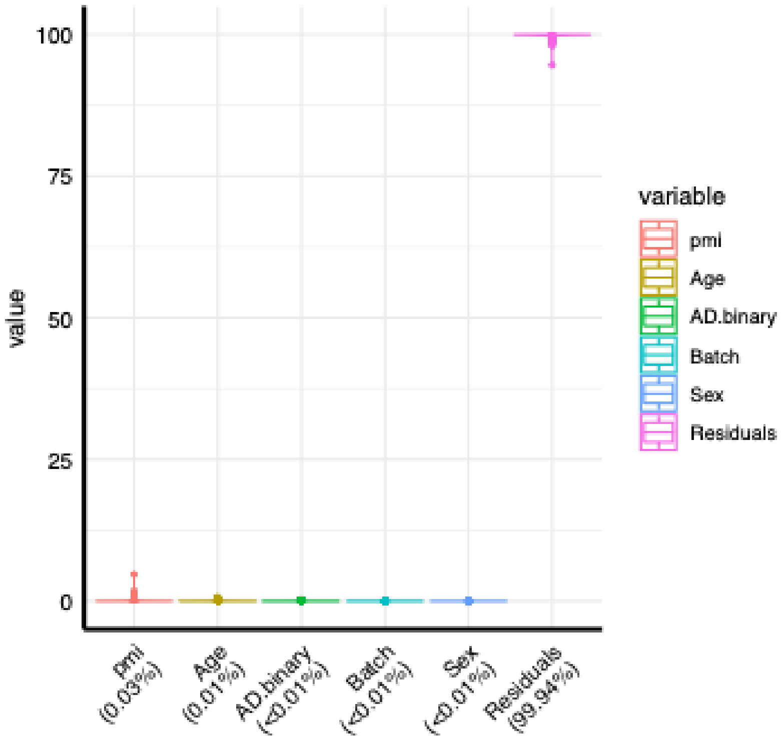

Supplement: Supplementary file 1 — Supplementary Notes A–C and Figs. 1–3. [file 41588_2025_2291_MOESM1_ESM.pdf]
