# Supplementary material for: Multiancestry brain pQTL fine-mapping and integration with genome-wide association studies of 21 neurologic and psychiatric conditions
Source: Nat Genet. 2025 Sep 8;57(9):2156–65. doi: 10.1038/s41588-025-02291-2 (PMC12425806; doi:10.1038/s41588-025-02291-2)
Supplement: Supplementary file 2 — Reporting Summary [file 41588_2025_2291_MOESM2_ESM.pdf]

nature portfolio

Corresponding author(s): Aliza P. Wingo, Thomas S. Wingo

Last updated by author(s): Jun 11, 2025

Reporting Summary

Nature Portfolio wishes to improve the reproducibility of the work that we publish. This form provides structure for consistency and transparency in reporting. For further information on Nature Portfolio policies, see our Editorial Policies and the Editorial Policy Checklist.

Statistics

For all statistical analyses, confirm that the following items are present in the figure legend, table legend, main text, or Methods section.

n/a	Confirmed	
☐	☒	The exact sample size (*n*) for each experimental group/condition, given as a discrete number and unit of measurement
☐	☒	A statement on whether measurements were taken from distinct samples or whether the same sample was measured repeatedly
☐	☒	The statistical test(s) used AND whether they are one- or two-sided<br>*Only common tests should be described solely by name; describe more complex techniques in the Methods section.*
☐	☒	A description of all covariates tested
☐	☒	A description of any assumptions or corrections, such as tests of normality and adjustment for multiple comparisons
☐	☒	A full description of the statistical parameters including central tendency (e.g. means) or other basic estimates (e.g. regression coefficient) AND variation (e.g. standard deviation) or associated estimates of uncertainty (e.g. confidence intervals)
☐	☒	For null hypothesis testing, the test statistic (e.g. *F*, *t*, *r*) with confidence intervals, effect sizes, degrees of freedom and *P* value noted<br>*Give P values as exact values whenever suitable.*
☒	☐	For Bayesian analysis, information on the choice of priors and Markov chain Monte Carlo settings
☒	☐	For hierarchical and complex designs, identification of the appropriate level for tests and full reporting of outcomes
☒	☐	Estimates of effect sizes (e.g. Cohen's *d*, Pearson's *r*), indicating how they were calculated

Our web collection on statistics for biologists contains articles on many of the points above.

Software and code

Policy information about availability of computer code

Data collection	An Orbitrap Fusion mass spectrometer (ThermoFischer Scientific) was used to collect raw proteomic data using Fusion software (version is 2.0.1258.15).
Data analysis	BioGRID database (v4.4.232 (April 28, 2024)), Bystro (v2.0.0-beta1), dplyr (v0.8.0.1), EIGENSOFT (v6.1.4), Fragpipe (v19.0), FUSION (commit e1ba5f7; https://github.com/gusevlab/fusion_twas), GEMMA (v0.98.1), ggplot2 (v3.1.0), GO-Elite (v1.2.5), KING (v2.2.2), MESuSiE (v1.0), MSFragger (v3.5), PEMapper/Pecaller (v2.0.0), Percolator (v3.0.5), Philosopher (v4.6.0), Plink (v1.90b), PMR (commit 7e49f14; https://github.com/yuanzhongshang/PMR), Post-MSFragger (v3.6), ProteinProphet (v4.6.0), qvalue (v2.15.0), R (v3.5.1), SMR (v1.02), susieR (v0.12.35), and SVA (v3.20.0).

For manuscripts utilizing custom algorithms or software that are central to the research but not yet described in published literature, software must be made available to editors and reviewers. We strongly encourage code deposition in a community repository (e.g. GitHub). See the Nature Portfolio guidelines for submitting code & software for further information.

Data

Policy information about availability of data

All manuscripts must include a data availability statement. This statement should provide the following information, where applicable:

- Accession codes, unique identifiers, or web links for publicly available datasets
- A description of any restrictions on data availability
- For clinical datasets or third party data, please ensure that the statement adheres to our policy

The data and results are deposited on Synapse ID syn64600176, https://doi.org/10.7303/syn64600176. These data include links to the raw and processed proteomic data, pQTL results, and protein weights from FUSION. These data and results are in whole or in part based on data obtained from the AMP-AD Knowledge Portal (https://adknowledgeportal.synapse.org/Explore/Programs/DetailsPage?Program=AMP-AD). The AD Knowledge Portal is a platform for accessing data, analyses and tools generated by the Accelerating Medicines Partnership (AMP-AD) Target Discovery Program and other National Institute on Aging (NIA)-supported programs to enable open-science practices and accelerate translational learning. The data, analyses and tools are shared early in the research cycle without a publication embargo on secondary use. Data are available for general research use according to the following requirements for data access and data attribution (https://adknowledgeportal.org/DataAccess/Instructions).

Research involving human participants, their data, or biological material

Policy information about studies with human participants or human data. See also policy information about sex, gender (identity/presentation), and sexual orientation and race, ethnicity and racism.

Reporting on sex and gender	The manuscript refers to sex in the biological sense of the word. As part of genotyping quality control, we checked the agreement between self reported gender and genetic sex.
Reporting on race, ethnicity, or other socially relevant groupings	All subjects with individual-level genetic data were divided into 3 populations based on self-report - African American (AA), Hispanic, and Non-Hispanic White (NHW). Within each self-report population, we remove outliers based on genetic ancestry by performing multidimensional scaling analysis (MDS) using their individual-level genetic data and Phase 3 1000 Genomes data as reference28. Self-defined Hispanic study participants were combined with the MXL (Mexican ancestry in Los Angeles, CA), PUR (Puerto Rican), CLM (Columbian), and PEL (Peruvian) reference panel populations. Self-defined NHW study participants were combined with the CEU (Utah Residents with Northern and Western European ancestry), TSI (Toscani in Italy), IBS (Iberian population in Spain), and FIN (Finnish) reference panel populations. Self-reported AA were combined with the ASW (African ancestry in Southwest USA) population.
Population characteristics	Post-mortem brain samples were collected by Rush Alzheimer's Disease Center, Mayo Clinic, Mount Sinai University Hospital, Emory University, and Brain and Body Donation Program at Banner Sun Health. For African American donors, the sample size was 181 with 63.5% (n=115) female and a mean age 79.3 years. For Hispanic/Latino American donors, the sample size 168 with 53.6% (n=90) females and a mean age was 77.1 years. For Non-Hispanic White donors, the sample size was 1,013 with 63.2% (n=640) female and a mean age of 87.6 years. Please see Table S1 for additional information about age of donors.
Recruitment	All research participants and post-mortem brain donors signed informed consent.
Ethics oversight	Our study complies with all relevant ethical regulations and approved by the Institutional Review Boards at Rush University, Mayo Clinic, Mount Sinai University, Emory University, Banner Sun Health Research Institute.

Note that full information on the approval of the study protocol must also be provided in the manuscript.

Field-specific reporting

Please select the one below that is the best fit for your research. If you are not sure, read the appropriate sections before making your selection.

☒ Life sciences ☐ Behavioural & social sciences ☐ Ecological, evolutionary & environmental sciences

For a reference copy of the document with all sections, see nature.com/documents/nr-reporting-summary-flat.pdf

Life sciences study design

All studies must disclose on these points even when the disclosure is negative.

Sample size	No sample size calculations were used. All available samples with brain proteomic, genetic, and phenotypic data were used for the analysis.
Data exclusions	utlier samples were removed in the quality control step of proteomic data. This was done through an iterative process of detecting outliers by principal component analysis of the proteomic data and excluding all individuals who were greater than 4 standard deviations from the mean of the first two principal components. Then we included individuals with both proteomic and genome-wide genotyping data for the analyses.
Replication	We examined the replication rate of brain pQTLs with prior published brain pQTLs and large published plasma pQTL datasets as provided in the results.
Randomization	For proteomic sequencing, samples were randomized to ensure even distribution of age, sex, PMI, cognitive diagnosis, and pathologies across

Randomization	sequencing batches.
Blinding	The individuals preparing samples for proteomic sequencing were blinded to phenotypic information.

Reporting for specific materials, systems and methods

We require information from authors about some types of materials, experimental systems and methods used in many studies. Here, indicate whether each material, system or method listed is relevant to your study. If you are not sure if a list item applies to your research, read the appropriate section before selecting a response.

Materials & experimental systems

n/a	Involved in the study	
☒	☐	Antibodies
☒	☐	Eukaryotic cell lines
☒	☐	Palaeontology and archaeology
☒	☐	Animals and other organisms
☒	☐	Clinical data
☒	☐	Dual use research of concern
☒	☐	Plants

Methods

n/a	Involved in the study	
☒	☐	ChIP-seq
☒	☐	Flow cytometry
☒	☐	MRI-based neuroimaging

Plants

Seed stocks	*Report on the source of all seed stocks or other plant material used. If applicable, state the seed stock centre and catalogue number. If plant specimens were collected from the field, describe the collection location, date and sampling procedures.*
Novel plant genotypes	*Describe the methods by which all novel plant genotypes were produced. This includes those generated by transgenic approaches, gene editing, chemical/radiation-based mutagenesis and hybridization. For transgenic lines, describe the transformation method, the number of independent lines analyzed and the generation upon which experiments were performed. For gene-edited lines, describe the editor used, the endogenous sequence targeted for editing, the targeting guide RNA sequence (if applicable) and how the editor was applied.*
Authentication	*Describe any authentication procedures for each seed stock used or novel genotype generated. Describe any experiments used to assess the effect of a mutation and, where applicable, how potential secondary effects (e.g. second site T-DNA insertions, mosiacism, off-target gene editing) were examined.*
